# Novel Subgroups in Subarachnoid Hemorrhage and Their Association With Outcomes—A Systematic Review and Meta-Regression

**DOI:** 10.3389/fnagi.2020.573454

**Published:** 2021-01-11

**Authors:** Ming-Dong Wang, Qian-Hui Fu, Ming-Jing Song, Wen-Bin Ma, John-H. Zhang, Zhan-Xiang Wang

**Affiliations:** ^1^Department of Neurosurgery, The First Affiliated Hospital of Xiamen University, School of Medicine, Xiamen University, Xiamen, China; ^2^School of Pharmacy, Minzu University of China, Beijing, China; ^3^Institute of Laboratory Animal Science, Peking Union Medical College, Chinese Academy of Medical Science, Beijing, China; ^4^Department of Neurosurgery, Peking Union Medical College Hospital, Chinese Academy of Medical Science, Beijing, China; ^5^Physiology Program, Department of Anesthesiology, Neurosurgery, Neurology, and Physiology, Center for Neuroscience Research, Loma Linda University School of Medicine, Loma Linda, CA, United States

**Keywords:** cohort studies, risk factors, subgroup subarachnoid hemorrhage, follow up risk time points, related-risk factors disease

## Abstract

**Background and Purpose:** Subarachnoid hemorrhage (SAH) has long been classified into two main forms, aneurysmal SAH (aSAH) and non-aneurysmal SAH (naSAH), but the related risk factors for aSAH and naSAH are heterogeneous. Our objective was to determine the risk factors for SAH of known or unknown origin with respect to diagnostic evaluation in a large patient cohort. We sought to determine whether our classification system can further predict middle long-term stroke and death.

**Methods:** We performed a systematic review and meta-analysis to identify risk factors for each SAH subtype. The discovery phase analyzed 11 risk factors from case studies in the literature. Kruskal-Wallis, Cox regression, logistic regression, and Kaplan-Meier analyses were used to compare the two groups.

**Results:** A total of 14,904 (34.53%) male and 22,801 (52.84%) female patients were eligible for this study. At a median follow-up of 45.6 months, the 5-years overall survival was 97.768% (95% CI: 0.259–0.292) for aSAH patients and 87.904% (95% CI: 1.459–1.643) for naSAH patients. The 10-years survival rate was 93.870% (95% CI: 2.075–3.086) and 78.115% (95% CI: 2.810–3.156), respectively. Multi-risk factor subgroups showed significant intergroup differences. We identified eight risk factors (drugs, trauma, neoplastic, vessels lesion, inflammatory lesion, blood disease, aneurysm, peri-mesencephalic hemorrhage) using logistic regression, which were optimally differentiated among the aSAH [aSAH-S (AUC: 1), a-d-SAH (AUC: 0.9998), aSAH-T (AUC: 0.9199), aSAH-N (AUC: 0.9433), aSAH-V (AUC: 1), aSAH-I (AUC: 0.9954), a-bd-SAH (AUC: 0.9955)] and naSAH [na-pmSAH (AUC: 0.9979), na-ni-ivl-SAH (AUC: 1), na-t-SAH (AUC: 0.9997), na-ne-SAH (AUC: 0.9475), na-d-SAH (AUC: 0.7676)] subgroups. These models were applied in a parallel cohort, showing eight risk factors plus survival rates to predict the prognosis of SAH.

**Conclusions:** The classification of risk factors related to aSAH and naSAH is helpful in the diagnosis and prediction of the prognosis of aSAH and naSAH patients. Further validation is needed in future clinical applications.

## Introduction

Spontaneous subarachnoid hemorrhage (SAH) was first described by Wilks in 1859 (Wilks, 1859). SAH accounts for 5% of strokes (Feigin et al., 2009), which refers to neurological dysfunction caused by bleeding from ruptured blood vessels to the cavity under arachnoid conditions. Clinical symptoms include abrupt onset, coma, recovery, and death and may be due to non-traumatic aneurysm hemorrhage, glioma, glioblastoma, arteriovenous angioma, atherosclerosis, dural thrombosis, etc. (Symonds, 1924; Russel and Kershman, 1937; Lin et al., 2006). In the early stage of SAH, non-contrast computed tomography (CT), a diagnostic test for SAH (Perry et al., 2011), and lumbar puncture supportive findings are essential requirements for effective intervention. In addition, angiography or autopsy is necessary for the diagnosis of the presence of a ruptured or an unbroken aneurysm. The incidence of SAH in patients with unruptured intracranial aneurysms (UIAs) is still unclear (Suarez et al., 2006) (residual aneurysm or unknown or not removed risk factors). Multiple risk factors, including age, location, size, multiple aneurysms, sex, accuracy of detection, environmental factors, lifestyle, and genetic factors, are associated with unruptured aneurysms, aneurysm formation, and death or disability in SAH patients (Macdonald and Schweizer, 2017). However, studies of risk factors have yielded some contradictory results that could be related to bias in the studies, which leads to a misunderstanding of aneurysms (Macdonald and Schweizer, 2017).

Currently, owing to the wide usage of diagnostic imaging approaches including magnetic resonance angiography (MRA) and computed tomography angiography (CTA), the initial diagnosis of occasional SAH has profoundly increased. The incidence rate of SAH is 2.0 per 100,000 people in China (Ingall et al., 2000), with a higher rate in women than in men (Shea et al., 2007; Van Munster et al., 2008; Sacco et al., 2009). Based on angiographic findings, SAH is categorized as either aneurysmal SAH (aSAH) or non-aneurysmal SAH (naSAH) (Mark, 2001; Raya et al., 2014); aSAH in 1 to 2% of patients is associated with trauma, infection, or tumor, and most intracranial ruptured aneurysms are small (Gijn et al., 2007)^1^. According to imaging detection of the blood distribution in the cavity of the subarachnoid, SAH could be described as peri-mesencephalic/prepontine (PM-SAH) or non-peri–mesencephalic hemorrhage (npm-SAH) in naSAH. In 2010, Nayak categorized four types of naSAH on the basis of CT findings and the position of bleeding (Nayak et al., 2010). However, these clinical imaging classification systems for SAH have not been integrated. It is difficult to use the variability of imaging and classification on the basis of a single factor to predict the prognosis. Furthermore, appropriate criteria are still lacking for the classification of naSAH and the scales to assess the prognosis of SAH patients. Thus, some researchers, including Fisher, Hijdra, Greene, and Morris and Marshall, designed a variety of classification schemes and attempted to establish a grading system based on the amount of subarachnoid bleeding in the subarachnoid cavity (Gijn et al., 2007).

To date, SAH research has been complicated due to the wide range of terms used to define different conditions, leading to confusion over potential pathophysiology. In addition, other risk factors, such as traumatic injury, hypertension, inflammation, blood disease, and neoplastic lesions, may contribute to SAH progression and recurrence. These factors are associated with an attributable risk of naSAH, aSAH, or aneurysm formation.

Many confounding risk factors make it difficult to classify a single type of SAH. This inconsistency in classification not only fails to improve the prognosis of patients but also may affect the pathological outcomes and even increase mortality. We proposed a refined classification approach with multiple axes based on digital subtraction angiography (DSA) and imaging (CTA and MRA). This approach also takes into account numerous factors or causes of either aSAH or naSAH, including the clinical symptoms of abrupt onset, recovery and pathophysiology, age, genetics, and follow-up. An ideal powerful classification tool would be able to identify the most important risk factors at diagnosis and guide clinicians in choosing an optimal personalized treatment, such as eliminating the aneurysm and its complications. Concurrently, we proposed a grading prognostic assessment system for SAH, which is a relatively new prognostic index for SAH patients. In this study, we used two statistical methods, multivariate Cox regression and Kaplan-Meier survival curves, to identify and weight the prognostic factors that are important to each subgroup.

## Methods

### Study Design and Search Strategy

This systematic review and meta-regression followed preferred reporting items for systematic reviews and meta-analyses (PRISMA) guidelines (Moher et al., 2009) and involved a systematic literature search of the MEDLINE databases to identify risk factors associated with SAH. In addition, priority was given to the commonly used quality assessment tools ROBIS (Risk of Bias in Systematic Reviews) and Quality Assessment of Diagnostic Accuracy Studies (QUADAS), which were taken into *a priori* consideration throughout the course of this study (Whiting et al., 2013, 2016).

We searched the MEDLINE database and combined the keywords used with Medical Subject Headings (MeSH) terms (see Appendix-total-1-the terms used in the search strategy and electronic databases, page 6 in Supplementary Material). The period of all original studies is from March 1, 1930 to April 1, 2019. The search terms were used to extract as many potentially relevant articles as possible from the database for the discovery of SAH-related risk factors. We also defined the selected and referenced optimal risk factors (see Appendix-total-1-page of 10 in Supplementary Material).

For risk factors, we have developed a set of theoretically reasonable methods and practices. These factors were explored in four stages to maintain as much homogeneity and methodological quality as possible. The first stages (the discovery stage/or identification and selection stage) reflected a mixture of risk factors in the longitudinal study. The second stage (identification of the best risk factors/or the stage of data collection and research evaluation) involved controlling confounding variables and finding the best risk factors for distinguishing between aSAH and naSAH cases. The third stage (disease progression stage/or synthesis and discovery stage) reflected the research on the relative risk of SAH to promote the occurrence and development of disease. The fourth stage (risk of bias in the review) refers to the concerns identified in different domains, with due consideration of the relevance of the search question and avoidance of excessive emphasis on the statistical significance of the results.

### Eligibility Criteria

To be eligible, the studies included in this systematic review and meta-regression stage were randomized controlled trials (RCTs), original cohort studies, and original case series/case-control studies. (1) The primary inclusion criterion for all studies was that the CT scans showed a confirmation of SAH and angiograms displayed the presence of intracranial aneurysm/no aneurysm, and only articles containing patient data were included in the systematic review. (2) Studies reporting cases of putative or alternative categories of risk factors associated with SAH were included. (3) The risk factors reported in all studies were not limited to only smoking or female sex and include trauma, drug use (cocaine, amphetamine, morphine, or antiepileptic drug use), inflammation, blood vessel disease, neoplastic lesions, blood disease, environmental factors, atherosclerosis, genetic factors, or hypertension. (4) In cohort and case/case-control studies, the inclusion criteria referred to people with a specific disease (the specific related diseases are summarized in Appendix-total-1 page 7 in Supplementary Material), and these studies were published in English or Chinese in a peer-reviewed journal or cited in a relevant journal article. (5) Studies had to use imaging for diagnosis with confirmed evidence of aneurysms in angiography, such as CT, CTA, MRA, or DSA, and had to be original research studies on adults with a history of spontaneous SAH. (6) The inclusion criteria for follow-up studies about the risk of rebleeding/death in patients after treatment were (1) patients with aneurysms who had previously undergone clipping/coil embolization and (2) all patients who had been followed up after being discharged and had undergone cerebral angiography at different times after surgery (each patient with aneurysms underwent related treatment during hospitalization and was followed up after discharge). (7) Cross-checking the references was performed until no new articles were identified. In this study, non-aneurysm peri-mesencephalic SAH (na-pmSAH) was divided into idiopathic patterns of peri-mesencephalic hemorrhage (IPH) and non-idiopathic patterns of peri-mesencephalic hemorrhage (Rinkel et al., 1991a,b).

### Exclusion Criteria

The following publications were excluded: (1) Articles classified as abstracts, comments/letters to the editor, reviews, lectures, and conference abstracts; (2) Those including patients with polycystic kidney disease, Ehlers-Danlos syndrome, Marfan syndrome, fibromuscular dysplasia, or Moyamoya syndrome; (3) Animal-based, model-based, and experimental studies; (4) Studies for which the full text is not available; (5) Basic research; and (6) Duplicate articles or data, poor imaging quality, and insufficient data for analysis research.

### Data Extraction and Quality Assessment

Two authors (WMD and MWB) independently reviewed all the titles and abstracts to assess their quantification. The included studies met the following requirements: (1) First, angiography was the basis for identifying aneurysm patients. We classified imaging methods as DSA or CTA/MRA. (2) The subtypes of each classification were based on angiography, and other lesions were detected by head magnetic resonance imaging, CT, histopathology, and urinary toxicology screening of patients. (3) Some subtypes were based on the patient's etiology combined with angiography. (4) On the basis of a different pathophysiology of each subtype, we subdivided the studies. (5) The Cochrane Collaboration's tool was used for assessing the quality and risk of bias in non-randomized intervention studies (cohort and case/case-control studies) and randomized controlled studies (ROBINS-1 see Appendix-total-1-page 9 in Supplementary Material) (Sterne et al., 2016). Differences were resolved through group discussions until a consensus was reached. We created an SAH classification system with causes and disease.

According to the inclusion and exclusion criteria, and to avoid data variability and follow-up heterogeneity, 43,151 SAH patients were finally enrolled in this study. These cases were randomly divided into a training cohort (aSAH = 16,687 and naSAH = 4,889), a testing cohort (aSAH = 6,674 and naSAH = 1,956), and a validation cohort (aSAH = 10,012 and naSAH = 2,933) with a ratio of 3–2:7–8 (please refer to Appendix 1-page 8).

### Clear Definition of Specifically Related Diseases

To accurately identify the population at increased risk of a disease or illness and analyze the distribution of different risk factors (specific) in different SAH patients, different risk factors were divided into two groups: aSAH and naSAH. We included blood disease, drugs, inflammatory diseases, neoplastic lesions, trauma, vessel diseases, non-inflammatory and cerebrovascular diseases, simple hemorrhage, and peri-mesencephalic hemorrhage as risk factors (see Appendix-total-1-pp10 in Supplementary Material). Although these different systemic diseases (as risk factors) are mixed, there is still a significant association between risk factors and SAH in the literature.

### Assessment of the Risk of Bias/Methodological Quality

According to the Cochrane Reviewers' Handbook for RCTs (Higgins and Green, 2011), two reviewers (WMD and FQH) independently performed the risk of bias assessment and the risk of bias tool for controlled trials and observational studies. The Cochrane Risk of Bias tool was used to assess the methodological quality of studies that met the inclusion criteria. The Risk of Bias assessment tool involved seven domains (please refer to Appendix 1-page 9 in Supplementary Material). We processed the information provided by the authors, including the name of author(s), publication year, type of clinical trial, sample size, age of participants, interventions, comparison groups, follow-up duration, outcomes, results, and conclusions. We also monitored the detailed information to support the risk of bias and assigned a judgement related to the risk of bias to this study. We completed a risk of bias table for each included study and presented the information graphically for all research areas (**Figures 3** and **4**).

### Statistical Analysis (Some Additional Notes on Statistics Appendix-Total-1 pp14)

GraphPad Prism version 6.01 software (GraphPad Software, La Jolla, CA, USA), R software (version 3.6.1), and SPSS-19 software (IBM SPSS Inc., Chicago, IL, USA) were used for all statistical analyses. A *P*-value of <0.05 was considered statistically significant. The relevant R language code package is included in the Supplementary Materials for reference (see Appendix-total-1-pp13 in Supplementary Material).

1. The meta-analysis for overall prevalence was conducted in the Stata version 10.0 (Stata Corp., College Station, TX, USA) and prepared in the R version 3.6.1 using the metaphor and meta packages (R Foundation for Statistical Computing. R Version 3.6.1, Vienna, Austria). Study heterogeneity was fully assessed by the chi-square test and *I*^2^ statistics. When *p* < 0.05 or *I*^2^ > 50%, a random-effects model was used.
2. Patient demographic and clinical characteristics were evaluated by using the chi-square test and t test for categorical variables and continuous variables, respectively.
3. Normally distributed continuous variables are expressed as the means ± standard error of the mean (SEM). Association of individual risk factors with SAH status was tested by using the Kruskal-Wallis test with *post-hoc* comparison by the Dunn test with Bonferroni correction. In the discovery phase, association of individual risk factors with SAH status was tested using the Kruskal-wallis test. Pairwise comparisons were then performed using the Dunn test with Bonferroni correction. These risk factors as variables and (tested for association with SAH status) used Meta-analysis to calculates pooled co-existing /associated ratios with 95% confidence interval (95%CI) and pooled odds ratios (ORs) with 95% CI for data. The statistical heterogeneity between specific OR by the I^2^ statistictest and Quantitative test, specificity yielded a P value of 0.05. For pooled data, the I^2^ statistic was used to estimate heterogeneity and risk of bias, specifically publication bias, based on the Egger test.
4. SAH are often complex and heterogeneous, and there may be varying related risk factors of overlap between two types, complicating an already intricate. For 9 risk factors aneurysmal-simple(aSAH-S), drugs (a-d-SAH & na-d-SAH), trauma (aSAH-T & na-t-SAH), neoplastic(aSAH-N & na-ne-SAH), vessels lesion(aSAH-V), Inflammatory lesion (aSAH-I), blood disease(a-bd-SAH), perimsencephalic haemorrhage (na-pmSAH), non-inflammatory & intracerebral vascular lesion(na-ni-ivl-SAH) were analyzed as Stepwise logistic regression (SLR) was used to find the risk factors associated with SAH that optimally distinguished between different risk factors. Multivariable analyses were performed by using the Cox proportional hazards (PH) regression models to assess the association between aSAH and the naSAH subgroup and the matched subgroups in aSAH and naSAH.
5. Using the sample function generates random numbers in the R software (version 3.6.1) and summarizes 50% of the data from the two sets of patient data sets as training data, 20% of the data as test data, and 30% of the data as a verification cohort (see Appendix 1-pp8). For each comparison, the data was randomly split into validation a-SAH and na-SAH. The SAH dataset was used to select variable and fit the model which was then tested on the validation set using receiver operating curve (ROC) analysis. The models developed for aSAH-S, a-d-SAH, aSAH-T, aSAH-N, aSAH-V, aSAH-I, and abd-SAH were test in the cases cohorts using ROC analysis. The models developed for na-pmSAH, na-ni-ivl-SAH, na-t-SAH, na-ne-SAH, and na-d-SAH were tested in the cohorts using ROC analysis.
6. Stepwise logistic regression was used to find the risk factors that best distinguished individuals who subsequently progressed from Pathogenic factors to Aneurysm formation from non-progressors. Stepwise selection was performed on the complete data, followed by ROC analysis with leave-one-out cross-validation. The diagnostic accuracy of aSAH and naSAH in SAH was evaluated using the receiver operating characteristic (ROC) curve analysis and logistic regression. The area under the curve (AUC) of ROC analysis, its 95% confidence interval and the statistical analysis were used to evaluate the diagnostic value of SAH classification; the OR value of logistic regression was used to further quantify. The confidence interval used in the Cochrane system review is 95%.
7. For survival analyses, we used the Kaplan-Meier method to analyze the correlation between variables and disease-free survival and the log-rank test to compare survival curves. The data were analyzed by the SPSS-19 software using the multiple logistic regression method. First, single factor logistic regression was used for the preliminary screening of risk factors. The factors with *P* < 0.10 in the univariate analysis were included in the multivariate logistic regression. *P* < 0.05 was considered statistically significant in multifactor analysis.

## Results

### Description of Included Studies

Figure A1 in the Supplementary Material shows the study selection process (Appendix-total-1, pp2 in Supplementary Material). After review, 933 articles were included in the meta-analysis. To establish a clinically available classification risk prediction model for different populations of SAH patients, among the 43,151 patients studied, 14,904 (34.53%) were male, 22,801 (52.84%) were female patients, and 5,446 (12.62%) lacked information about sex (Table 1). The median follow-up periods of 1, 2, and 5 years were 6, 8.85, and 45.6 months, respectively (range 0–11.33 years). The 3-months to 5-years follow-up numbers (FNs) for the aSAH and naSAH cohorts (*n* = 10,665, *n* = 3,759) were 31.96 and 38.44%, respectively. The 5- to 12-years FN rates for the aSAH and naSAH cohorts (*n* = 5,812, *n* = 660) were 36.23 and 14.94%, respectively. A total of 159 (0.44%) patients were lost to follow-up. SAH recurrence occurred during the follow-up period, affecting 38 (0.114%-aSAH) and four (0.041%-naSAH) patients. Moreover, rebleeding from the aneurysm occurred. The incidence of SAH recurrence was one in 878-aSAH (2,439-naSAH) patient-years. In the aSAH group, there were 647 (1.94%) deaths, 115 (0.34%) autopsy reports (death certificates), and 354 (3.62%) deaths from naSAH.

**Figure 1:**
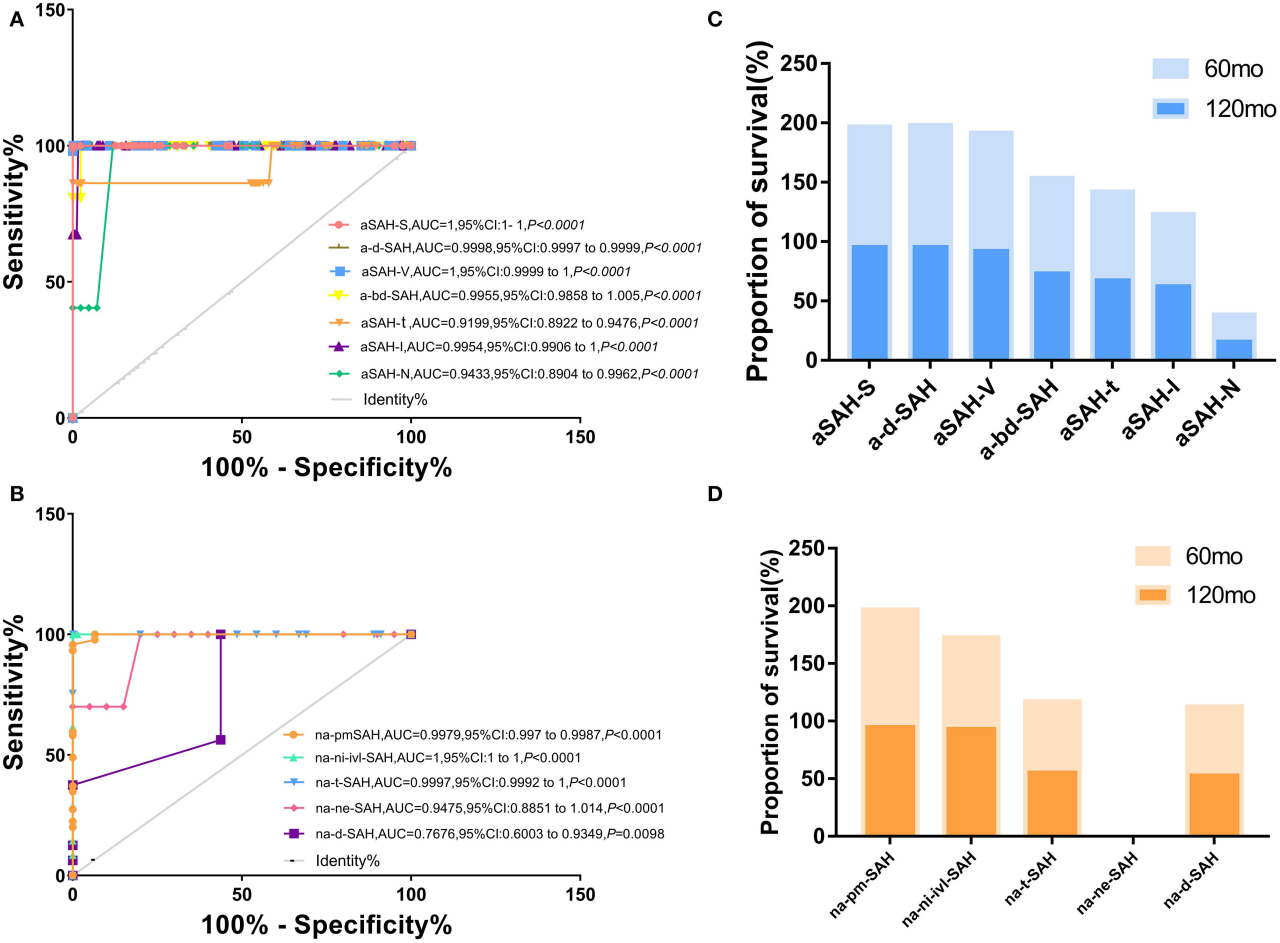
Different risk factors combined with clinical SAH improve accuracy of predicting models for a-SAH, na-SAH. **(A,B)** Receiver operator characteristic (ROC) curves for different risk factors along in a-SAH subtype **(A)** and na-SAH subtype **(B)**. The value the AUC (the area under the ROC curve) and 95% CI. **(A)** Receiver operator characteristic (ROC) curves for different risk factors along in a-SAH subtype. ROC curve showing the sensitivity and specificity of a-SAH subgroup classification. **(B)** Receiver operator characteristic (ROC) curves for different risk factors along in na-SAH subtype. ROC curve showing the sensitivity and specificity of na-SAH subgroup classification. **(C)** Survival ratio for individual a-SAH subgroup categorized based on follow-up time after 60 and 120 months. Significant difference in a-SAH subgroup. **(D)** Survival ratio for individual na-SAH subgroup categorized based on follow-up time after 60 and 120 months. Significant difference in na-SAH subgroup.

**Table 1:** Meta-analysis of SAH for subgroups and baseline and disease characteristics stratified by aneurysm status.

**Characteristics**	**Aneurysmal-SAH (aSAH)**							**Non-aneurysmal-SAH (naSAH)**				
	**aSAH-S (*n* = 21,909)**	**a-d-SAH (*n* = 7,562)**	**aSAH-T (*n* = 290)**	**aSAH-N (*n* = 154)**	**aSAH-V (*n* = 2,335)**	**aSAH-I (*n* = 826)**	**a-bdSAH (*n* = 297)**	**na-pm-SAH (*n* = 3,249)**	**na-d-SAH (*n* = 47)**	**na-ne-SAH (*n* = 79)**	**na-t-SAH (*n* = 2,310)**	**na-ni-ivl-SAH (*n* = 4,093)**
**Sex (%)**												
Male	6,687 (30.52%)	2,382 (31.50%)	190 (65.52%)	64 (41.55%	751 (32.16%)	365 (44.19%)	87 (29.29%)	1,507 (46.38%)	16 (34.04%)	38 (48.10%)	1,302 (56.36%)	1,515 (37.01%)
Female	13,072 (59.66%)	5,109 (67.56%)	99 (34.14%)	90 (58.44%)	636 (23.24%)	369 (44.67%)	192 (64.65%)	1,362 (41.92%)	10 (21.27%)	31 (39.24%)	732 (31.69%)	1,099 (26.85%)
L G	2,150 (9.81%)	71 (0.94%)	1 (0.34%)	0	948 (40.60%)	92 (11.14%)	18 (6.06%)	380 (11.70%)	21 (44.68%)	10 (12.66%)	276 (11.95%)	1,479 (36.13%)
Random effects 95%CI	0.28 (0.22, 0.33)	0.50 (0.38, 0.63)	0.26 (0.20, 0.32)	0.35 (0.29, 0.41)	0.45 (0.33, 0.58)	0.28 (0.23, 0.32)	0.63 (0.50, 0.75)	0.49 (0.38, 0.61)	0.67 (0.45, 0.90)	0.38 (0.24, 0.52)	0.36 (0.25, 0.48)	0.10 (0.09, 0.11)
Fixed effects 95%CI	0.09 (0.09, 0.09)	0.20 (0.20, 0.20)	0.07 (0.06, 0.08)	0.16 (0.13, 0.18)	0.50 (0.50, 0.58)	0.13 (0.13, 0.14)	0.13 (0.12, 0.14)	0.63 (0.63, 0.64)	0.58 (0.51, 0.65)	0.41 (0.36, 0.46)	0.36 (0.35, 0.37)	0.01 (0.01, 0.01)
I^2^**/τ**^2^	100/0.671	100/0.2310	97/0.0527	74/0.0393	100/0.2789	98/0.0832	98/0.1214	100/0.1693	89/0.1523	85/0.1043	99/0.0885	99/0.0020
Heterogeneity *P***-**value	0	0	0	<0.01	0	0	<0.01	0	<0.01	<0.01	0	0
**Follow-up time (%)**												
3–12 mo	1,635 (7.46%)	1,895 (25.06%)	172 (59.31%)	15 (9.74%)	46 (1.97%)	13 (1.78%)	15 (5.05%)	529 (16.28%)	15 (31.91%)	16 (20.25%)	490 (21.21%)	937 (22.89%)
1–3 years	1,466 (6.69%)	31 (0.41%)	50 (17.24%)	11 (7.14%)	407 (17.43%)	111 (15.26%)	12 (4.04%)	225 (6.93%)	1 (2.13%)	3 (3.80%)	114 (4.93%)	801 (19.57%)
3–5 years	1,616 (7.38%)	2,769 (36.62%)	4 (1.38%)	14 (9.09%)	320 (13.70%)	49 (5.93%)	14 (4.71%)	75 (2.31%)		1 (1.27%)	199 (8.61%)	353 (8.62%)
5–8 years	5,215 (23.80%)	0		1 (0.65%)	141 (6.04%)	20 (2.42%)					18 (0.77%)	145 (3.54%)
8–12 years	255 (1.16%)	0		1 (0.65%)	124 (5.31%)	54 (10.26%)	1 (0.36%)	208 (6.40%)			81 (3.50%)	208 (5.08%)
Recurrence	38 (0.17%)											4 (0.10%)
Died/(+FD)	392 (1.79%)/(343+49)	83 (1.10%)	31 (10.69%)/(28 + 3)	25 (16.23%)	20 (0.86%)	81 (9.81%)/(75 + 6)	15 (5.05%)	68 (2.09%)/(48 + 20)	10 (21.28%)	21 (24.56%)/(13 + 8)	221 (9.56%)	34 (0.83%)
Autopsied	105 (0.48%)	10 (0.13%)										
Uncertain	11,100 (50.66%)	2,774 (36.68%)	33 (11.38%)	87 (56.49%)	1,277 (54.69)	489 (59.20%)	220 (74.07%)	2,082 (53.00%)	30 (63.83%)	38 (48.10%)	1,186 (51.34%)	
Lost follow-up	87 (0.4%)					9 (1.71%)		62 (2.54%)			1 (0.04%)	

*CI, confidence interval; FD, follow-up period died; mo, months; a-SAH-S, aneurysmal Subarachnoid hemorrhage-simple; a-d-SAH, aneurysmal-drugs subarachnoid hemorrhage; a-SAH-T, aneurysmal Subarachnoid hemorrhage co-existing trauma; a-SAH-N, aneurysmal Subarachnoid hemorrhage co-existing neoplastic; a-SAH-V, aneurysmal Subarachnoid hemorrhage co-existing vesselas lesion; a-SAH-I, aneurysmal Subarachnoid hemorrhage co-existing with Inflammatory lesion; a-bd-SAH, aneurysmal-blood disease subarachnoid hemorrhage; LG, lack of description of gender; CI, confidence interval; HR, hazard ratio*.

### Stratification of SAH-Related Risk Factors at Different Stages of Discovery (Literature Search Results)

The literature search yielded 27,447 SAH articles, including 23,478 aSAH and 3,969 naSAH articles. Among them, 933 articles (aSAH-634, naSAH-299) were identified in the discovery databases (these basically cover all years/coverage from 1930 to 2018). The factor is the online-only data supplement file (statistical analysis AND stratification of SAH-related risk factors). The distribution of the risk factors and discovery is displayed and a group comparison is shown in Table 2. Among the 12 subgroups (risk factors) analyzed categorically, the clinical subgroups showed statistically significant differences.

**Table 2:** Twelve (nine risk factors) subgroup analysis associated with clinical state in the discovery phase.

**Variable**	**a-SAH**	***P*****-value**		***P*****-value**		***P*****-value**		***P*****-value**		***P*****-value**		***P*****-value**	
	**Mean ± SD**	**aSAH-S vs. a-d-SAH**		**aSAH-S vs. aSAH-T**		**aSAH-S vs. aSAH-N**		**aSAH-S vs. aSAH-V**		**aSAH-S vs. aSAH I**		**aSAH-S vs. a-bd-SAH**	
		**KW**	**D-t**	**KW**	**D-t**	**KW**	**D-t**	**KW**	**D-t**	**KW**	**D-t**	**KW**	**D-t**
aSAH-S	214.79 ± 551.31	0.000017	NS	0.000001	0.004	0.000001	0.004	0.000001	0.022	0.000001	0.004	0.000001	0.005
aSAH-T	3.76 ± 14.62		NS		NS		NS		0.005		NS		NS
aSAH-N	1.65 ± 1.91		NS		NS		NS		0.001		0.007		NS
aSAH-V	31.13 ± 59.38		0.024		0.005		0.001		NS		0.005		0.040
aSAH-I	4.32 ± 9.73		NS		NS		0.007		0.005		NS		NS
a-d-SAH	130.37 ± 651.08		NS		NS		NS		NS		NS		NS
a-bd-SAH	7.07 ± 20.65		NS		NS		NS		0.048		NS		NS
**Variable**	**na-SAH**	***P*****-value**			***P*****-value**			***P*****-value**			***P*****-value**		
	**Mean** **±** **SD**	**na-pmSAH vs. na-d-SAH**			**na-pmSAH vs. na-ne-SAH**			**na-pmSAH vs. na-t-SAH**			**na-pmSAH vs. na-ni-ivlSAH**		
		**KW**		**D-t**	**KW**		**D-t**	**KW**		**D-t**	**KW**		**D-t**
na-pmSAH	63.70 ± 52.55	0.00001		0.000	0.0001		0.000	0.588		NS	0.00001		0.044
na-d-SAH	4.53 ± 5.61			NS			0.13			0.000			0.000
na-ne-SAH	2.92 ± 3.51			NS			0.11			0.000			0.000
na-t-SAH	85.55 ± 117.24			0.13			0.11			0.096			NS
na-ni-ivl-SAH	22.92 ± 47.95			0.000			0.000			0.096			0.044

*KW, kurskal-Wallis; NS, not significant; D-t, Dunn test; SD, standard deviation; na-pmSAH, non-aneurysmal perimsencephalic hemorrhage; na-ni-ivl-SAH, non-aneurysmal and non- inflammatory and intracerebral vascular lesion subarachnoid hemorrhage; na-t-SAH, non-aneurysmal-traumatic subarachnoid hemorrhage; na-ne-SAH, non-aneurysmal-neoplastic subarachnoid hemorrhage; na-d-SAH, non-aneurysmal-drugs subarachnoid hemorrhage. Showed statistically significant difference in clinical subgroup. a-SAH-S, aneurysmal Subarachnoid hemorrhage-simple; a-d-SAH, aneurysmal-drugs subarachnoid hemorrhage; a-SAH-T, aneurysmal Subarachnoid hemorrhage co-existing trauma; a-SAH-N, aneurysmal Subarachnoid hemorrhage co-existing neoplastic; a-SAH-V, aneurysmal Subarachnoid hemorrhage co-existing vesselas lesion; a-SAH-I, aneurysmal Subarachnoid hemorrhage co-existing with Inflammatory lesion; a-bd-SAH, aneurysmal-blood disease subarachnoid hemorrhage. Showed statistically significant difference in clinical subgroup*.

The weighted incidence rate of aSAH-S from 102 studies was 15.02% (Appendix-2.1 Figure S1 in Supplementary Material). According to the aSAH subcomponent level, the incidence rates were 8.39% for a-d-SAH, 11.34% for aSAH-T, 13.69% for aSAH-N, 11.05% for aSAH-V, 28.13% for aSAH-I, and 6.19% for a-bd-SAH. Among the na-pmSAH subgroup, the incidence rate was 16.78% for na-pmSAH. The incidence rate was 60.53% for na-ni-ivl-SAH, 8.89% for na-t-SAH, 8.89% for na-ne-SAH, and 4.93% for na-d-SAH (Appendix-2.1, 2.2 Figures S1–12 in Supplementary Material, Table 1).

### Relative Risk of SAH in Related Risk Factor Disease

When aSAH and naSAH groups were compared in a *post-hoc* exploratory SAH group analysis, aSAH and naSAH with various subgroups did not differ significantly. After adjusting for stratification direction (analysis and comparison between subgroups), significant improvements were observed in the aSAH [HR 2.316 (95% CI 1.770–3.032); *P* < 0.0001] vs. naSAH [HR 0.432 (95% CI 0.330–0.565) *P* < 0.0001] groups. The two-way comparison between aSAH-S/na-pmSAH vs. a-d-SAH/na-d-SAH, aSAH-T/na-t-SAH, aSAH-N/na-ne-SAH, aSAH-V, aSAH-I, and a-bd-SAH/na-ni-ivl-SAH subgroups is shown in Table 3. These findings indicate that various subgroups are relatively independent of risk factors.

**Table 3:** Multivariate model for hazard ratio (HR) of categories risk factors in aSAH and naSAH subgroup vs. the matched subgroup within aSAH and naSAH.

	**Intra group**				**Intra group**		
	**Hazard ratio**	***p*-Value**	**95%CI**		**Hazard ratio**	***p*-Value**	**95%CI**
a-SAH-S	15.739	<0.0001	12.040–20.573	a-d-SAH	15.091	<0.0001	10.240–22.241
	4.171	<0.0001	3.184–5.465	aSAH-T	0.049	<0.0001	0.033–0.072
	4.304	<0.0001	3.294–5.624	aSAH-N	0.036	<0.0001	0.024–0.055
	5.618	<0.0001	4.295–7.350	aSAH-V	2.541	<0.0001	1.574–4.102
	3.028	<0.0001	2.278–4.026	aSAH-I	0.075	<0.0001	0.056–0.100
	4.705	<0.0001	3.613–6.126	a-bd-SAH	0.116	<0.0001	0.057–0.236
na-pmSAH	0.067	<0.0001	0.045–0.100	na-t-SAH	0.063	<0.0001	0.046–0.086
	0.297	<0.0001	0.220–0.403	na-ne-SAH	0.133	<0.0001	0.059–0.300
	0.295	<0.0001	0.218–0.399	na-d-SAH	0.052	<0.0001	0.024–0.111
	1.113	0.488	0.823–1.504	na-ni-ivl-SAH	22.976	<0.0001	16.004–32.985
	**Matched group**				**Matched group**		
	**Hazard ratio**	***p*****-Value**	**95%CI**		**Hazard ratio**	***p*****-Value**	**95%CI**
a-SAH-T	35.221	<0.0001	23.722–52.292	a-d-SAH	0.469	<0.0001	0.321–0.685
	46.029	<0.0001	31.062–68.207	aSAH-N	63.485	<0.0001	41.604–41.604
	41.027	<0.0001	27.708–60.747	aSAH-V	1.046	<0.0001	0.641–1.705
	59.070	<0.0001	39.560–88.202	aSAH-I	29.360	<0.0001	22.272–38.705
	42.690	<0.0001	28.846–63.179	a-bd-SAH	21.081	<0.0001	10.393–42.760
a-d-SAH	2.238	<0.0001	1.534–3.266	aSAH-N	47.571	<0.0001	31.215–72.498
	2.519	<0.0001	1.728–3.673	aSAH-V	1.251	<0.0001	0.773–2.024
	1.764	0.0004	1.201–2.592	aSAH-I	0.045	<0.0001	0.034–0.060
	2.397	<0.0001	1.645–3.493	a-bd-SAH	0.065	<0.0001	0.032–0.132
aSAH-N	0.018	<0.0001	0.012–0.028	aSAH-V	0.934	0.780	0.580–1.504
	0.012	<0.0001	0.008–0.018	aSAH-I	0.035	<0.0001	0.027–0.046
	0.018	<0.0001	0.012–0.027	a-bd-SAH	0.050	<0.0001	0.025–0.102
aSAH-V	0.612	0.038	0.385–0.973	aSAH-I	0.039	<0.0001	0.029–0.051
	1.046	0.875	0.644–1.698	a-bd-SAH	0.056	<0.0001	0.028–0.113
aSAH-I	0.040	<0.0001	0.020–0.081	a-bd-SAH	0.039	<0.0001	0.030–0.051
na-ni-ivl-SAH	0.390	0.0001	0.219–0.695	na-t-SAH	0.064	<0.0001	0.036–0.112
	3.303	<0.0001	2.608–4.183	na-ne-SAH	0.264	0.0001	0.117–0.595
	3.281	<0.0001	2.590–4.155	na-d-SAH	0.103	<0.0001	0.048–0.220
na-t-SAH	0.131	<0.0001	0.104–0.166	na-ne-SAH	0.063	<0.0001	0.028–0.144
	0.131	<0.0001	0.104–0.166	na-d-SAH	0.024	<0.0001	0.011–0.053
na-ne-SAH	0.154	<0.0001	0.069–0.346	na-d-SAH	0.062	<0.0001	0.029–0.131

*CI, confidence interval; a-SAH-S, aneurysmal Subarachnoid hemorrhage-simple; a-d-SAH, aneurysmal-drugs subarachnoid hemorrhage; a-SAH-T, aneurysmal Subarachnoid hemorrhage co-existing trauma; a-SAH-N, aneurysmal Subarachnoid hemorrhage co-existing neoplastic; a-SAH-V, aneurysmal Subarachnoid hemorrhage co-existing vesselas lesion; a-SAH-I, aneurysmal Subarachnoid hemorrhage co-existing with Inflammatory lesion; a-bd-SAH, aneurysmal-blood disease subarachnoid hemorrhage; na-pmSAH, non-aneurysmal perimsencephalic hemorrhage; na-ni-ivl-SAH, non-aneurysmal and non-inflammatory and intracerebral vascular lesion subarachnoid hemorrhage; na-t-SAH, non-aneurysmal-traumatic subarachnoid hemorrhage; na-ne-SAH, non-aneurysmal-neoplastic subarachnoid hemorrhage; na-d-SAH, non-aneurysmal-drugs subarachnoid hemorrhage*.

### Developing Models to Differentiate Stratified Risk Factors (SAH From aSAH and naSAH)

#### aSAH From aSAH-S, a-d-SAH, aSAH-T, aSAH-N, aSAH-V, aSAH-I, and a-bd-SAH

Stepwise selection of aSAH from aSAH-S, a-d-SAH, aSAH-T, aSAH-N, aSAH-V, aSAH-I, and a-bd-SAH showed an interdependence between certain risk factors related to SAH and revealed significant and independent high-risk factors for SAH that help distinguish clinical subgroups. We incorporated aSAH-S, a-d-SAH, aSAH-T, aSAH-N, aSAH-V, aSAH-I, and a-bd-SAH models. The diagnostic accuracy in distinguishing aSAH-S, a-d-SAH, aSAH-T, aSAH-N, aSAH-V, aSAH-I, and a-bd-SAH from aSAH was high (univariate ROC analysis AUC: 1, 0.9998, 0.9199, 0.9433, 1, 0.9954, and 0.9955). We correctly predicted 89.89% of samples with a sensitivity of 75.48% and a specificity of 94.55% (Figure 1A). We further evaluated its performance in distinguishing aSAH subgroups.

#### naSAH Model Composed of na-d-SAH, na-ne-SAH, na-t-SAH, na-ni-ivi-SAH, and na-pmSAH

The naSAH model composed of na-d-SAH, na-ne-SAH, na-t-SAH, na-ni-ivi-SAH, and na-pmSAH correctly predicted 70.84% of the samples with a sensitivity of 90.86% and a specificity of 72.57%. Furthermore, the accuracy of replicating models in na-d-SAH, na-ne-SAH, na-t-SAH, na-ni-ivi-SAH, and na-pmSAH was high (univariate ROC analysis AUC: 0.767, 0.9475, 0.9997, 1, and 0.9979) (Figure 1B). These findings suggest that this classification system based on the risk factors that distinguished between aSAH and naSAH was reliable and feasible. We further evaluated its performance in distinguishing subgroups of non-aneurysmal subarachnoid hemorrhage.

### Comparison of Survival Rates and Possible Death and Rebleeding Events at Different Follow-Up Risk Time Points in Each Risk Subgroup (SAH Grade Prognostic Assessment)

The 5-years survival rate [97.768% (95% CI: 0.292–0.259)] of aSAH was significantly higher than that of naSAH [87.904% (95% CI: 1.459–1.643)]. Additionally, the 10-years survival rate (93.870% [95% CI: 2.075–3.086)] was significantly higher than that of naSAH [78.115% (95% CI: 2.810–3.156)].

**Figure 2:**
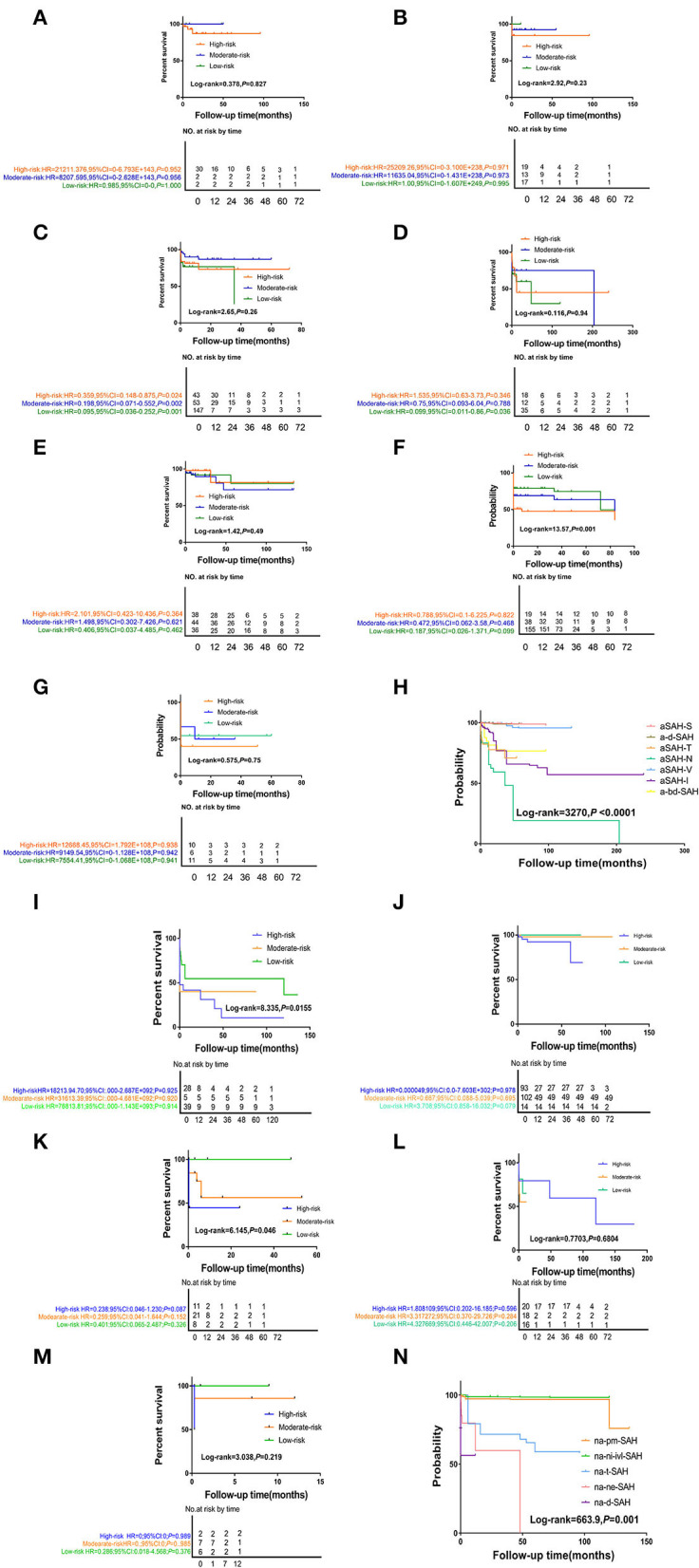
The Kaplan-Meier survival curve analysis of differ follow-up timepoint for SAH subtype stratified by disease-related or probable causes. (1) **(A)** the aSAH-S subgroup, **(B)** the a-d-SAH subgroup, **(C)** the aSAH-T subgroup, **(D)** the aSAH-N subgroup, **(E)** the aSAH-V subgroup, **(F)** the aSAH-I subgroup, **(G)** the a-bd-SAH subgroup, **(H)** aSAH-subgroup total figure. (2) **(I)** the na-pmSAH subgroup, **(J)** the na-ni-ivl-SAH subgroup, **(K)** the na-t-SAH subgroup, **(L)** the na-ne-SAH subgroup, **(M)** the na-d-SAH subgroup, **(N)** naSAH-subgroup total figure.

The short-term survival rates of patients with naSAH and aSAH was significantly higher than the long-term survival rate. Compared with the naSAH subgroups, the aSAH subgroups showed more significant differences in the overall survival (OS) and follow-up period associated with different risk factors (Figure 2). In addition, we evaluated the possibility of rebleeding or death events associated with different follow-up periods using improved SR assessment by an exploratory Kaplan-Meier survival ratio (SR) analysis. The outcomes showed that different follow-up times may represent different survival risk levels. In the aSAH-S subgroup and other subgroups, patients with low, intermediate, and high risk had different risk levels at different follow-up times, with SRs of 99.695, 99.695, 99.439% [2 months: HR 0.985 (95% CI 0–0) for low risk, 2 months: HR 8207.595 (95% CI 0–2.628) for moderate, and 30 months: HR 21211.376 (95% CI 0–6.793) for high risk] (Figure 2-(1), Tables 4, 5).

**Table 4:** The risk levels and survival rates of aneurysmal subarachnoid hemorrhage from differ follow-up timepoint.

**DTP: RL**	**aSAH-S**		**a-d-SAH**		**aSAH-T**		**aSAH-N**		**aSAH-V**		**aSAH-I**		**a-bd-SAH**	
	**SR**	**HR**	**SR**	**HR**	**SR**	**HR**	**SR**	**HR**	**SR**	**HR**	**SR**	**HR**	**SR**	**HR**
2 mo: L	99.695% (0.090–0.128)	0.985 (0–0)	99.893% (0.062–0.149)		85.186% (5.001–7.221)		83.333% (8.349–15.140)		99.805% (0.146–0.582)		97.580% (1.325–2.886)		87.804% (6.929–14.654)	
2 mo: IM	–	8207.595 (0–2.628)	–		–		–		–		–		–	
30 mo: H	99.439% (0.135–0.178)	21211.376 (0–6.793)	98.965% (0.309–0.440)		77.742% (16.872–9.317)		59.240% (14.078–17.896)		99.576% (0.256–0.705)		76.938% (4.766–5.772)		76.737% (11.319–19.111)	
13 mo: IM	99.695% (0.090–0.128)		99.6018% (0.167–0.291)	11635.04 (0–1.431)	93.112% (3.296–6.112)		65.476% (12.987–17.737)		99.697% (0.205–0.634)		92.338% (2.704–4.086)		81.533% (9.290–16.679)	
17 mo: L	–		99.358% (0.230–0.359)	1.00 (0–1.607)	–		62.358% (13.566–17.863)		–		91.935% (2.785–4.156)		81.533% (9.290–16.679)	
19 mo: H	–		98.965% (0.309–0.440)	25209.26 (0–3.1)	–		59.240% (14.078–17.896)		99.576% (0.265–0.705)		85.080% (3.875–5.075)		–	
43 mo: H	99.439% (0.135–0.178)		98.965% (0.309–0.440)		70.981% (9.831–13.371)	0.395 (0.148–0.875)	48.132% (15.869–17.956)		97.584% (1.002–1.697)		65.848% (6.261–7.215)		76.737% (11.319–19.111)	
53 mo: IM	99.149% (0.178–0.225)		–		70.981 (9.831–13.371)	0.198 (0.071–0.0552)	19.253% (12.822–17.938)		96.140% (1.435–2.257)		–		–	
147 mo: L	97.803 (0.999–1.816)		–			0.059 (0.036–0.252)	–		95.794% (1.537–2.392)		57.121% (7.655–8.553)		–	
12 mo: IM	99.695% (0.090–0.128)		99.608% (0.167–0.291)		77.742% (16.872–9.317)		65.476% (12.987–17.237)	0.75 (0.093–3.73)	99.697% (3.250–5.467)		92.741% (2.622–4.015)		81.533% (17.568–24.491)	
18 mo: H	–		98.965% (0.309–0.440)		–		62.358% (13.566–17.863)	1.535 (0.63–3.73)	–		91.129% (2.938–4.288)		–	
35 mo: L	99.439% (0.135–0.178)		–		70.981% (9.831–13.371)		59.240% (14.078–17.896)	0.099 (0.011–0.86)	99.408% (0.350–0.854)		76.938% (4.766–5.772)		76.737% (11.319–19.111)	
36 mo: L	99.439% (0.135–0.178)		98.965% (0.309–0.440)		70.981% (9.831–13.371)		48.132% (15.869–17.956)		99.408% (0.350–0.854)	0.406 (0.037–4.485)	76.938% (4.766–5.772)		76.737% (11.319–19.111)	
38 mo: H	–		–		–		–		97.584% (1.002–1.697)	2.101 (0.423–10.43 6)	65.848% (6.261–7.215)		–	
44 mo:IM	–		–		–		–		97.584% (1.002–1.697)	1.498 (0.302–7.426)	–		–	
19 mo: H	99.695% (0.090–0.128)		98.965% (0.309–0.440)		70.981% (9.831–13.371)		54.405% (13.726–16.330)		99.576% (0.265–0.705)		85.080% (3.875–5.075)	0.788 (0.1–6.225)	81.533% (9.290–16.679)	
38 mo:IM	99.439% (0.135–0.187)		–		–		–		97.584% (1.002–1.697)		65.848% (6.261–7.215)	0.472 (0.062–3.58)	76.737% (11.319–19.111)	
155 mo: L	97.803% (0.999–1.816)		–		–		21.762% (34.359–19.977)		95.794% (1.537–2.392)		57.121% (7.655–8.553)	0.187 (0.026–1.371)	–	
6 mo: IM	99.695% (0.090–0.128)		99.893% (0.062–0.149)		82.315% (5.609–7.810)		67.836% (10.430–13.749)		99.805% (0.146–0.582)		95.564% (1.954–3.430)		87.804% (6.929–14.654)	9149.54 (0–1.128)
10 mo: H	–		99.608% (0.167–0.291)		–		64.605% (11.362–14.666)		–		94.758% (2.164–3.614)		84.668% (8.183–15.833)	12668.45 (0–1.792)
11 mo: L	–		–		–		61.205 (12.268–15.452)		–		–		–	7554.41 (0–1.068)

*mo, months; CI, confidence interval; L, low; IM, intermediate; H, high; HR, hazard ratio; SR, survival ratio; “–”, ditto; DTP, differ time point; RL, risk level; a-SAH-S, aneurysmal Subarachnoid hemorrhage-simple; a-SAH-d, aneurysmal-drugs subarachnoid hemorrhage; a-SAH-T, aneurysmal Subarachnoid hemorrhage co-existing trauma; a-SAH-N, aneurysmal Subarachnoid hemorrhage co-existing neoplastic; a-SAH-V, aneurysmal Subarachnoid hemorrhage co-existing vesselas lesion; a-SAH-I, aneurysmal Subarachnoid hemorrhage co-existing with Inflammatory lesion; a-bd-SAH, aneurysmal-blood disease subarachnoid hemorrhage*.

**Table 5:** The risk levels and survival ratio characteristics of patients with non-aneurysmal subarachnoid hemorrhage.

**DTP: RL**	**na-pmSAH**		**na-ni-ivl-SAH**		**na-t-SAH**		**na-ne-SAH**		**na-d-SAH**	
	**SR**	**HR**	**SR**	**HR**	**SR**	**HR**	**SR**	**HR**	**SR**	**HR**
3 mo: IM	98.812% (0.511–0.893)	21985.72 (0.0–6.262)	99.959% (0.035–0.249)		79.224% (2.781–3.145)		79.687% (12.173–25.205)		56.25% (19.973–26.707)	
5 mo: L	97.211% (0.966–1.468)	67571.49 (0.0–1.936)	98.772% (0.367–0.523)		–		–		–	
49 mo: H	96.793% (1.158–1.797)	13128.7 (0.0–3.758)	98.501% (0.457–0.656)		67.921% (3.685–4.031)		0% (24.974–30.779)		0	
13 mo: H	97.211% (0.966–1.468)		98.772% (0.367–0.523)	0.916 (0–1.999)	79.224% (2.781–3.145)		59.765% (25.628–41.092)		0	
85 mo: L	96.793% (1.158–1.797)		98.222% (0.626–0.961)	5715.663 (0–2.823)	58.880% (5.079–5.500)		0		0	
87 mo:IM	–		98.222% (0.626–0.961)	1144.758 (0–5.620)	–		0		0	
8 mo: L	97.211% (0.966–1.468)		98.772% (0.367–0.523)		79.224% (2.781–3.145)	0.401 (0.065–2.478)	79.687% (12.173–25.205)		56.25% (19.973–26.707)	
11 mo: H	–		–		79.224% (2.781–3.145)	0.238 (0.046–1.230)	79.687% (12.173–25.205)		–	
21 mo: IM	–		–		71.522% (3.429–23.851)	0.259 (0.046–1.644)	59.765% (25.628–41.092)		0	
7 mo: IM	97.211% (0.966–1.468)		98.772% (0.367–0.523)		79.224% (2.781–3.145)		79.687% (12.173–25.205)	2.986 (0.332–26.855)	56.25% (19.973–26.707)	
13 mo: L	–		–		–		59.765% (25.628–41.092)	4.194 (0.429–41.012)	0	
18 mo: H	–		–		71.522% (3.429–23.851)		59.765% (25.628–41.092)	1.381 (0.086–22.214)	0	
2 mo: H	98.812% (0.511–0.893)		98.772% (0.367–0.523)		79.224% (2.781–3.145)		79.687% (12.173–25.205)		56.25% (19.973–26.707)	0 (0)
6 mo: L	97.211% (0.966–1.468)		–		–		–		56.25% (19.973–26.707)	52.020 (0.018–4.568)
7 mo: IM			–		–		–		56.25% (19.973–26.707)	0 (0)

*mo, months; L, low; IM, intermediate; H, high; HR, hazard ratio; SR, survival ratio; “–”, ditto; DTP, differ time point; RL, risk level; na-pmSAH, non-aneurysmal perimsencephalic hemorrhage; na-ni-ivl-SAH, non-aneurysmal and non- inflammatory and intracerebral vascular lesion subarachnoid hemorrhage; na-t-SAH, non-aneurysmal-traumatic subarachnoid hemorrhage; na-ne-SAH, non-aneurysmal-neoplastic subarachnoid hemorrhage; na-d-SAH, non-aneurysmal-drugs subarachnoid hemorrhage*.

Interestingly, the effect of follow-up periods between high-, intermediate-, and low-risk groups was heterogeneous, although the interaction was significantly different (HR = 67,571.49, 95% CI: 0.0–1.936). This showed that there were obvious differences in their correlation (HR = 21,985.72, 95% CI: 0.0–6.262 vs. HR = 13,128.70, 95% CI: 0.0–3.758, *P* = 0.0–3.758) (Figure 2-(2), Table 5). For patients with rebleeding or death events, close monitoring is necessary to identify the rebleeding and death happening at high-risk time points. On the other hand, patients in the na-pmSAH subgroup showed a 13-months SR of 97.211% (95% CI: 0.511–0.893) and a 30-months SR of 98.641% (95% CI: 0.966–1.468).

SR was the highest in aSAH-S (93.803%) in the aSAH subgroup and na-pm-SAH (98.222%) in the naSAH subgroup. At 10 years, aSAH-N had the lowest SR (19.253%). Among the different SAH subgroups, the longest SR was aSAH-S (97.803%), followed by ad-SAH&V (93.62 and 95.794%, respectively) and a-bd-SAH (76.737%) (Figures 1C,D). Due to the lack of 5- and 10-years follow-up, the histogram of na-ne-SAH was missed.

### Risk of Bias (Methodological Quality Assessment)

**Figure 3:**
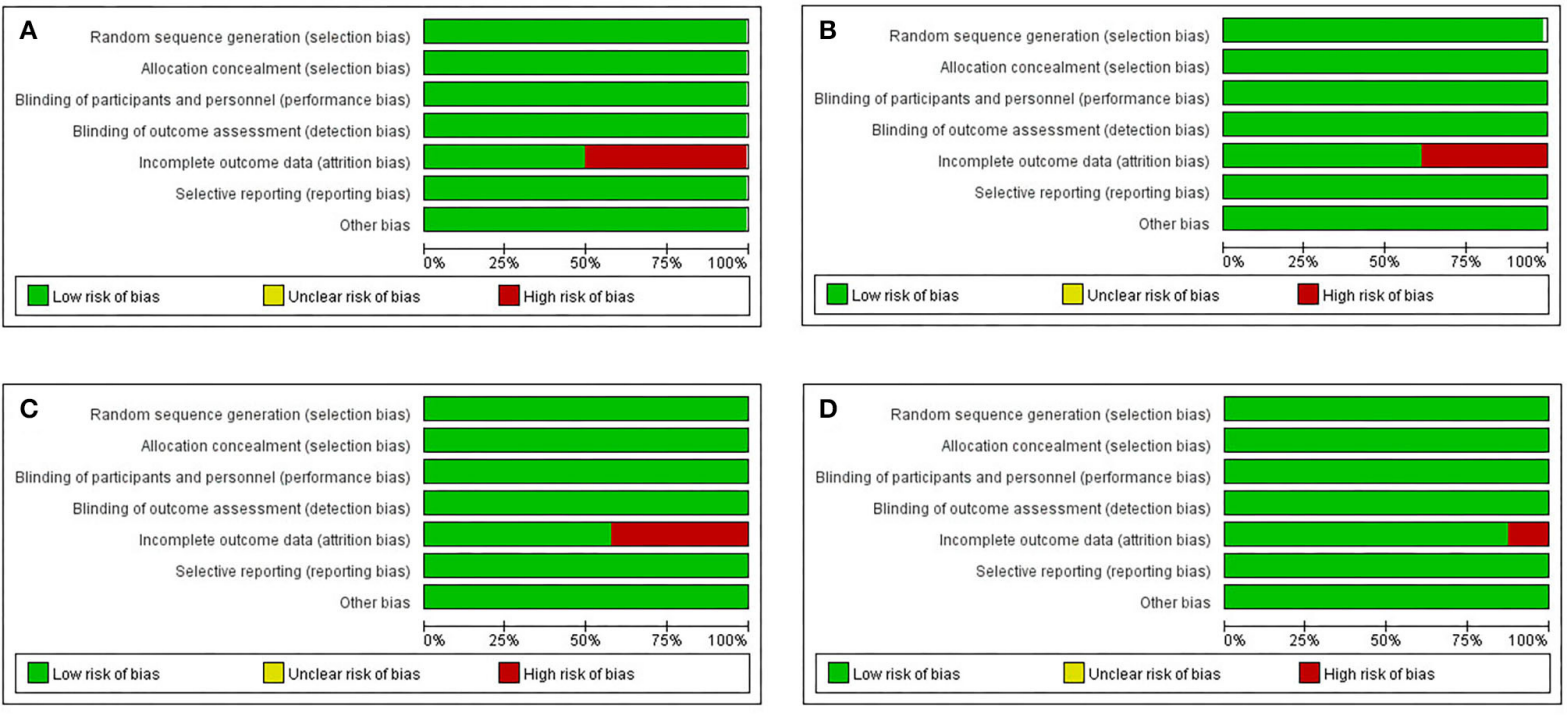
Risk of bias and applicability concerns graph: review author's judgements about each risk of bias item for each include randomized controlled study (RCT), cohort original studies and case series/case-control original studies (domain presented as percentages across included studies). **(A)** Case-control original studies, **(B)** Case-cohort original studies, **(C)** Case series report studies, **(D)** RCT.

**Figure 4:**
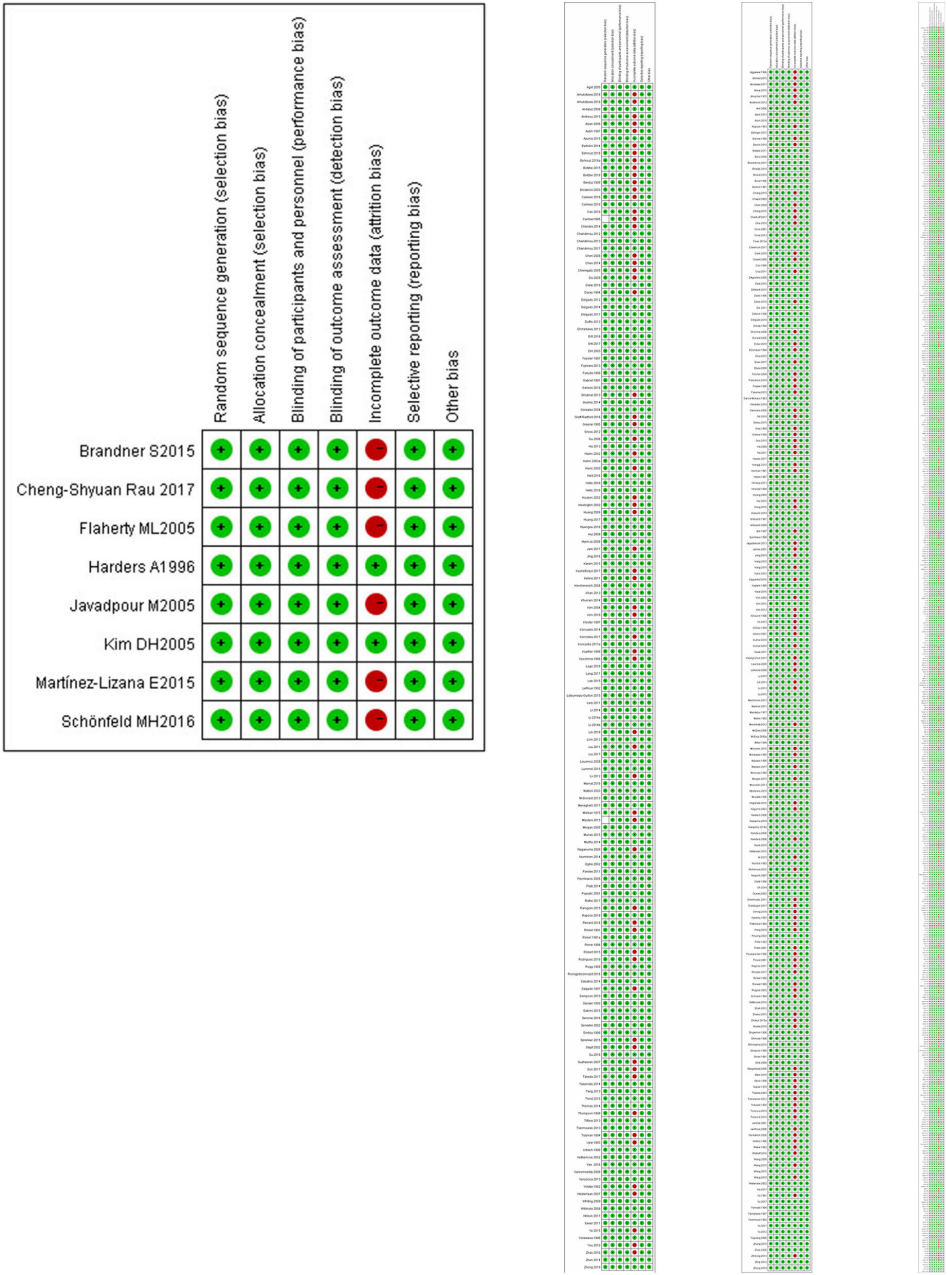
Risk of bias and applicability concerns summary: review author's judgements about each risk of bias item for each included RCT.

Figure 3 shows the risk of bias, including RCTs (0.09%), original cohort studies (17.59%), and original case series/case-control studies (82.41%) (see Appendix-3.1, 3.2, 3.3 in Supplementary Material). The bias risk of all the research literature was evaluated in seven domains, including eight RCT studies, 565 studies (case series), 198 studies (case control), and 162 studies (case cohort), in the absence of incomplete outcome data (attrition bias). The risk of attrition bias was considered low (Figure 4), and in terms of measurement bias, only one study in the case series was at serious risk.

## Discussion

This new approach for categorizing subgroups of SAH patients is superior to the traditional method for SAH patient classification. The approach described in the current study can detect high-risk factors for aneurysmal and non-aneurysmal SAH at the time of diagnosis and help explore major risk factors involved in the etiology, early diagnosis, stratification, and prediction of stroke course. It also provides information necessary for guiding treatment preferences. The current classification is neither suitable for screening individuals on the basis of major risk factors nor for identifying related types of diseases or assessing responses to interventions.

The nine risk factors were markedly different between the aSAH and naSAH subgroups of different clinical conditions. Step-by-step selection demonstrated that the risk factors related to the formation of intracranial aneurysms were highly associated with the risk factors related to SAH. However, some risk factors significantly and independently help distinguish SAH clinical subgroups. The analysis displayed the best model for aSAH-S vs. aSAH subtypes, including a-d-SAH/aSAH-T/aSAH-N/aSAH-V/aSAH-I/a-bd-SAH. The a-d-SAH and aSAH-I subgroup statuses showed AUCs of 0.9998 and 0.9954 in the discovery cohort and were considered to be highly predictive in demonstrating the clinical specific features, course of related diseases, and follow-up examination of seven subgroups. The data showed that the overall and subgroup survival rates of aSAH patients relative to naSAH patients were significantly higher than those of high-risk stroke patients. Nevertheless, one may argue that diagnoses of patients with aneurysmal hemorrhage based on CT findings could be indicators of poor prognosis. Recurrences occurred in the aSAH-S and na-ni-ivl-SAH subgroups during the 1.4-years follow-up period (Serrone et al., 2016). Furthermore, rebleeding occurred at the target aneurysm. Elderly patients older than 60 years of age experienced recurrence during the follow-up period.

UIAs should be classified as the aSAH-S subtype, and the number of patients identified to have UIAs would increase over time. DSA or CTA data support the hypothesis that non-na-pmSAH is decreased given that it is unclear whether non-na-pmSAH is truly caused by microaneurysm. Therefore, it is necessary to emphasize that the current simple types can be used only as a basis for further classification. A model analysis includes data and disease types. Arteriovenous malformations (AVMs) are a congenital vascular abnormality that occurs predominantly in young patients (Dalton et al., 2018). They are still an independent risk factor for intracranial aneurysm formation (Juvela et al., 2001), but 636 (47.99%) patients in the aSAH-V subgroup were found to be female. In the aSAH-I subgroup, inflammatory diseases that cause an aneurysm were identified, including Lyme borreliosis (LB), intracranial mycotic aneurysms (IMA), Behçet's disease (BD), and polyarteritis nodosa (PAN) (Polet and Weinstein, 1999; Ducruet et al., 2010; Gupta et al., 2013; Ha et al., 2016; John et al., 2016; Sangwoo et al., 2016). Intracerebral and/or intraventricular hemorrhage in patients with leukemia is a common cause of mortality. Sickle-cell anemia (SCA), Diamond–Blackfan anemia (DBA), idiopathic thrombocytopenic purpura (ITP), afibrinogenemia, and acute lymphoblastic leukemia belong to the a-bd-SAH subgroup (Trivedi et al., 2002; Vicari et al., 2004; Aggarwal et al., 2013; Kassim et al., 2016).

Distinguishing the diagnosis of non-aneurysmal subarachnoid hemorrhage based on bleeding on CT and angiography-negative subarachnoid hemorrhage is still controversial (Rinkel et al., 1991a). Among currently reported naSAH patients, patients with idiopathic peri-mesencephalic hemorrhage have a good prognosis. However, we still know very little about naSAH and non-na-peri-mesencephalic SAH. We tried to validate our subgroup system in the naSAH cohort.

The best disease models were tested in naSAH cohorts, which included 9,778 patients: na-ni-ivl-SAH subgroup (AUC 1), na-pmSAH, na-ne-SAH, na-t-SAH (AUC: 0.9979, 0.9475, 0.9997), and na-d-SAH subgroup (AUC: 0.767). These models were validated in a multi-clinical center cohort study, indicating that these naSAH subgroup classifications can facilitate diagnosis and differential diagnosis and may provide a basis for clinical practice. In previous studies, the na-pmSAH subgroup was characterized only by blood distribution of the blood in the subarachnoid space anterior to the midbrain or the pons, which had a lower rate of vasospasm (Kang et al., 2009; Gross et al., 2012). Patients with IPH (local clots in the peri-mesencephalic cisterns) have a good prognosis, while patients with indistinguishable blood distribution have rebleeds from aneurysm hemorrhage confirmed on CT. Although recent data have shown an increasing trend of na-pm-SAH, it is difficult to estimate the incidence of na-pmSAH in naSAH (Konczalla et al., 2016a,b; Lago et al., 2016). Through clinical meta-analysis, we can preliminarily conclude that the incidence of the na-pmSAH subgroup in naSAH is 16.78%. Based on the follow-up data analyzed by the Kaplan-Meier analysis, the risk of rebleeding or death at different time points was divided into high, moderate, and low risk, and the strength of this association varied between high, intermediate, and low risk. Risk factors for hemorrhagic disease in non-aneurysmal and non-inflammatory vascular disease (na-ni-ivl-SAH), including carotid artery dissection (CAD), dural arteriovenous fistulas (DAVF), cerebral amyloid angiopathy (CAA), and cerebral venous thrombosis (CVT) (Lin et al., 2006; Mendel et al., 2017). The radiological features of na-ni-ivl-SAH predominantly show cortical sulci and convex hemorrhage on CT, and the aneurysm is negative in contrast.

Through our analysis and review of the subject, we can conclude that different types of aSAH and naSAH have the same relatively independent conditional risk factors. Trauma (aSAH-T) is the most common cause of an aneurysm, such as a middle meningeal artery aneurysm (Maekawa et al., 2009). In addition to a direct correlation between na-t-SAH and the incidence of hypoxia, hypotension, skull fractures, brain contusions, and intracranial hypertension, trauma is also one of the most important independent negative prognostic factors of the abovementioned cases (Mata-Mbemba et al., 2015, 2018). na-t-SAH and aSAH-T may exhibit different bleeding patterns on CT, but there may be significant tentorial SAH in the former (Connolly et al., 2012; Perry et al., 2015). Intra-cavernous aneurysms (IAs) (aSAH-N) may coexist with PAs, meningiomas, glioma, retinoblastoma, acoustic neurilemmoma, RCC, and cerebral malformations (Raskind, 1965; Gonzales-Portillo and Valdivia, 2006; Vogel et al., 2011; Akutsu et al., 2014). Although na-ne-SAH is the rarest type of SAH, it occurs due to bleeding from vascularization of the pituitary apoplexy (PA), cerebral metastases (CM), malignant glioma (MG), acoustic neuroma (AN), schwannoma of the cranial nerve, cervical meningiomas, cervical spinal cord hemangioblastoma, and spinal meningeal carcinomatosis with negative angiography (Gliemroth et al., 1999; Garg et al., 2004; Javalkar et al., 2009; Khanna et al., 2013; Heit et al., 2017). Drug-related SAH is relevant to cocaine abuse, and no aneurysms are shown or present during angiography (Rinkel et al., 1991a). The source of bleeding in patients without an aneurysm is unknown (Gliemroth et al., 1999). Although biopsy-proven vasculitis has been found in patients with cocaine abuse (Krendel et al., 1990), na-d-SAH related to the use of anticoagulant drugs is rare. The aneurysm is not found on angiography (Levine et al., 1991).

At this stage, we cannot claim that the new subgroups represent different causes of SAH or aneurysm, nor can we say that this classification is the best classification of SAH subtypes. It is possible to further stratify by other variables (such as genotype or other risk factors). This study is retrospective, and multicenter data should have variability and increased clinical follow-up heterogeneity, which also affects the tracking statistical models. However, the authors searched multiple databases, which may lead to selection bias. These results may indicate the possibility of delayed publication bias. Over time, with updated research, evidence suggests that multiple factors contribute to the formation of aneurysms. In addition, aneurysms may not have been detected in some patients due to the lack of technological progress or angiography in studies published before 1980. Therefore, the formation, location, number, and proximity of aneurysms cannot be determined.

In conclusion, information from some variables that are critical to the development of SAH or aneurysm is superior for estimating modifiable risk factors (smoking, drinking, exercise, serum cholesterol, and hypertension). Our exploratory subgroup research shows that it is necessary to extend clinical observation data to clinical practice to find more evidence. Clinically useful stratification represents an important step in stroke precision medicine.

## Ethics Statement

The ethics committee at the First Affiliated Hospital of Xiamen University, School of Medicine, Xiamen University approved the study protocol (Approval No. KYX-2019-012).

## Author Contributions

M-DW and J-HZ: conception and design. M-DW, Z-XW, M-JS, and W-BM: acquisition of data. M-DW and M-JS: analysis and interpretation of data. M-DW and W-BM: drafting the article. J-HZ and Z-XW: critically revising the article and approved the final version of the manuscript on behalf of all authors. M-DW, M-JS, and Q-HF: statistical analysis. Z-XW, J-HZ, and W-BM: administrative/technical/material support and study supervision. All authors reviewed the submitted version of manuscript.

## Conflict of Interest

The authors declare that the research was conducted in the absence of any commercial or financial relationships that could be construed as a potential conflict of interest.

## Footnotes

^1^Youman's neurology surgery, p1476, p3772, p3740. Chapter 361, p3747, p3428. Chapter 361 p3662, p3752.

## Abbreviations

a-bd-SAH: aneurysmal-blood disease subarachnoid hemorrhage
a-d-SAH: aneurysmal drug subarachnoid hemorrhage (cocaine abuse)
aSAH-I: aneurysmal subarachnoid hemorrhage coexisting with inflammatory lesion
aSAH-N: aneurysmal subarachnoid hemorrhage coexisting with neoplasm
aSAH-S: aneurysmal subarachnoid hemorrhage-simple
aSAH-T: aneurysmal subarachnoid hemorrhage with coexisting trauma
aSAH-V: aneurysmal subarachnoid hemorrhage coexisting with blood vessel lesion
na-d-SAH: non-aneurysmal drug subarachnoid hemorrhage
na-ne-SAH: non-aneurysmal-neoplastic subarachnoid hemorrhage
na-ni-ivl-SAH: non-aneurysmal, non-inflammatory and intracerebral vascular lesion subarachnoid hemorrhage
na-pmSAH: non-aneurysmal peri-mesencephalic hemorrhage
na-t-SAH: non-aneurysmal traumatic subarachnoid hemorrhage.

## References

[B1] Aggarwal A. Salunke P. Mathuriya S. N. Futane S. (2013). Intraventricular hemorrhage in a patient with chronic myeloid leukemia and anterior communicating artery aneurysm. Neurol. India 61, 308–310. 10.4103/0028-3886.115076 23860156

[B2] Akutsu N. Hosoda K. Ohta K. Tanaka H. Taniguchi M. Kohmura E. (2014). Subarachnoid hemorrhage due to rupture of an intracavernous carotid artery aneurysm coexisting with a prolactinoma under cabergoline treatment. J. Neurol. Surg. Rep. 75, e73–e76. 10.1055/s-0033-1364166 25083394 PMC4110122

[B3] Connolly E. S. Jr, Rabinstein, A. A. Carhuapoma J. R. Derdeyn C. P. Dion J. Higashida R. T.. (2012). Guidelines for the management of aneurysmal subarachnoid hemorrhage. Stroke 43, 1711–1737. 10.1161/STR.0b013e3182587839 22556195

[B4] Dalton A. Dobson G. Prasad M. Mukerji N. (2018). De novo intracerebral arteriovenous malformations and a review of the theories of their formation. Br. J. Neurosurg. 32, 305–311. 10.1080/02688697.2018.1478060 29873271

[B5] Ducruet A. F. Hickman Z. L. Zacharia B. E. Narula R. Grobelny B. T. Gorski J.. (2010). Intracranial infectious aneurysms: a comprehensive review. Neurosurg. Rev. 33, 37–46. 10.1007/s10143-009-0233-1 19838745

[B6] Feigin V. L. Lawes C. M. Bennett D. A. Barker-Collo S. L. Parag V. (2009). Worldwide stroke incidence and early case fatality reported in 56 population–based studies: a systematic review. Lancet Neurol. 8, 355–369. 10.1016/S1474-4422(09)70025-0 19233729

[B7] Garg A. Chugh M. Gaikwad S. B. Chandra S. P. Gupta V. Mishra N. K.. (2004). Juvenile pilocytic astrocytoma presenting with subarachnoid hemorrhage. Case report and review of the literature. J. Neurosurg. 100(5 Suppl Pediatrics), 525–529. 10.3171/ped.2004.100.5.0525 15287467

[B8] Gijn J. van Kerr Richard S. Rinkel Gabriel J. E. (2007). Subarachnoid haemorrhage. Lancet 369, 306–318. 10.1016/S0140-6736(07)60153-6 17258671

[B9] Gliemroth J. Nowak G. Kehler U. Arold H. (1999). Neoplastic cerebral aneurysm from metastatic lung adenocarcinoma associated with cerebral thrombosis and recurrent subarachnoid haemorrhage. J. Neurol. Neurosurg. Psychiatry 66, 246–247. 10.1136/jnnp.66.2.246 10071112 PMC1736208

[B10] Gonzales-Portillo G. A. Valdivia V. J. M. (2006). Uncommon presentation of pediatric ruptured intracranial aneurysm after radiotherapy for retinoblastoma. Case report. Surg. Neurol. 65, 391–396. 10.1016/j.surneu.2005.07.074 16531206

[B11] Gross B. A. Lin N. Frerichs K. U. Du R. (2012). Vasospasm after spontaneous angiographically negative subarachnoid hemorrhage. Acta Neurochir. 154, 1127–1133. 10.1007/s00701-012-1383-4 22588341

[B12] Gupta V. Chinchure S. D. Goel G. Jha A. N. Malviya S. Gupta R. (2013). Coil embolization of intracranial aneurysm in polyarteritis nodosa. A case report and review of the literature. Interv. Neuroradiol. 19, 203–208. 10.1177/159101991301900209 23693044 PMC3670059

[B13] Ha S. Kim J. Kim C. Jang S. J. (2016). Multiple intracranial aneurysms associated with Behçet's disease. J. Cerebrovasc. Endovasc. Neurosurg. 18, 32–37. 10.7461/jcen.2016.18.1.32 27114964 PMC4842906

[B14] Heit J. J. Iv M. Wintermark M. (2017). Imaging of intracranial Hemorrhage. J. Stroke 19, 11–27. 10.5853/jos.2016.00563 28030895 PMC5307932

[B15] Higgins J. P. T. Green S. (2011). Cochrane Handbook for Systematic Reviews of Interventions. London: John Wiley & Sons.

[B16] Ingall T. Asplund K. Mahonen M. Bonita R. (2000). A multinational comparison of subarachnoid hemorrhage epidemiology in the WHO MONICA stroke study. Stroke 31, 1054–1061. 10.1161/01.str.31.5.1054 10797165

[B17] Javalkar V. Guthikonda B. Vannemreddy P. Nanda A. (2009). Association of meningioma and intracranial aneurysm: report of five cases and review of literature. Neurol. India 57, 772–776. 10.4103/0028-3886.59475 20139508

[B18] John S. Walsh M. K. Ferdinand K. H. Sundararajan S. Silverman S. Mark B. (2016). Dynamic angiographic nature of cerebral mycotic aneurysms in patients with infective endocarditis. Stroke. 47, e8–e10. 10.1161/STROKEAHA.115.011198 26604253

[B19] Juvela S. Poussa K. Porras M. (2001). Factors affecting formation and growth of intracranial aneurysms: a long-term follow-up study. Stroke 32, 485–491. 10.1161/01.STR.32.2.485 11157187

[B20] Kang D. H. Park J. Lee S. H. Park S. H. Kim Y. S. Hamm I. S. (2009). Does non-perimesencephalic type non-aneurysmal subarachnoid hemorrhage have a benign prognosis? J. Clin. Neurosci. 16, 904–908. 10.1016/j.jocn.2008.10.008 19362482

[B21] Kassim A. A. Pruthi S. Day M. Rodeghier M. Gindville M. C. Brodsky M. A.. (2016). Silent cerebral infarcts and cerebral aneurysms are prevalent in adults with sickle cell anemia. Blood 127, 2038–2040. 10.1182/blood-2016-01-694562 26941400

[B22] Khanna A. Venteicher A. S. Walcott B. P. Kahle K. T. Mordes D. A. William C. M.. (2013). Glioblastoma mimicking an arteriovenous malformation. Front. Neurol. 4:144. 10.3389/fneur.2013.00144 24137154 PMC3786388

[B23] Konczalla J. Kashefiolasl S. Brawanski N. Lescher S. Senft C. Platz J.. (2016b). Cerebral vasospasm and delayed cerebral infarctions in 225 patients with non-aneurysmalsubarachnoid hemorrhage: the underestimated risk of Fisher 3 blood distribution. J. Neurointerv. Surg. 8, 1247–1252. 10.1136/neurintsurg-2015-012153 26847333

[B24] Konczalla J. Schmitz J. Kashefiolasl S. Senft C. Platz J. Seifert V. (2016a). Non-aneurysmal non-perimesencephalic subarachnoid hemorrhage: effect of rehabilitation at short-term and in a prospective study of long-term follow-up. Top Stroke Rehabil. 23, 261–268. 10.1080/10749357.2016.1149982 26916565

[B25] Krendel D. A. Ditter S. M. Frankel M. R. Ross W. K. (1990). Biopsy proven cerebral vasculitis associated with cocaine abuse. Neurology. 40, 1092–1094. 10.1212/wnl.40.7.1092 2356010

[B26] Lago A. López-Cuevas R. Tembl J. I. Fortea G. Górriz D. Aparici F.. (2016). Short- and long-term outcomes in non-aneurysmal non-perimesence phalic subarachnoid hemorrhage. Neurol Res. 38, 692–697. 10.1080/01616412.2016.1200306 27338138

[B27] Levine S. R. Brust J. C. M. Futrell N. Brass L. M. Blake D. Fayad P.. (1991). A comparative study of the cerebrovascular complications of cocaine: alkaloidal versus hydrochloride. A review. Neurology 41, 1173–1177. 10.1212/WNL.41.8.1173 1866000

[B28] Lin J. H. Kwan S. Y. Wu D. (2006). Cerebral venous thrombosis initially presenting with acute subarachnoid hemorrhage. J. Chin. Med. Assoc. 69, 282–285. 10.1016/S1726-4901(09)70258-8 16863016

[B29] Macdonald R. L. Schweizer T. A. (2017). Spontaneous subarachnoid haemorrage. Lancet 398, 655–666. 10.1016/S0140-6736(16)30668-7 27637674

[B30] Maekawa H. Tanaka M. Hadeishi H. (2009). Middle meningeal artery aneurysm associated with meningioma. Acta. Neurochir. 151, 1167–1168. 10.1007/s00701-009-0263-z 19319475

[B31] Mark S. G. (2001). Handbook of Neurosurgery. Stuttgart; New York, NY: Thieme p. 754.

[B32] Mata-Mbemba D. Mugikura S. Nakagawa A. Murata T. Ishii K. Kushimoto S. (2018). Traumatic midline subarachnoid hemorrhage on initial computed tomography as a marker of severe diffuse axonal injury. J. Neurosurg. 5, 1–8. 10.3171/2017.6.JNS17466 29303451

[B33] Mata-Mbemba D. Mugikura S. Nakagawa A. Murata T. Kato Y. Tatewaki Y.. (2015). Intraventricular hemorrhage on initial computed tomography as marker of diffuse axonal injury after traumatic brain injury. J. Neurotrauma 32, 359–365. 10.1089/neu.2014.3453 25026366

[B34] Mendel T. A. Błazejewska-Hyzorek B. Szpak G. M. Stepień T. Lewandowska E. Tarka S.. (2017). Intracerebral hemorrhage in the context of cerebral amyloid angiopathy and varied time of onset of cerebral venous thrombosis: a case report. Folia Neuropathol. 55, 242–248. 10.5114/fn.2017.70490 28984118

[B35] Moher D. Liberati A. Tetzlaff J. Altman D. G. PRISMA Group (2009). Preferred reporting items for systematic reviews and meta-analyses: the PRISMA statement. J. Clin. Epidemiol. 62, 1006–1012. 10.1016/j.jclinepi.2009.06.005 19631508

[B36] Nayak S. Kunz A. B. Kieslinger K. Ladurner G. Killer M. (2010).Classification of non-aneurysmal subarachnoid haemorrhage: CT correlation to the clinical outcome. Clin. Radiol. 65:e623e628. 10.1016/j.crad.2010.01.022 20599064

[B37] Perry J. J. Alyahya B. Sivilotti M. L. Bullard M. J. Émond M. Sutherland J.. (2015). Differentiation between traumatic tap and aneurysmal subarachnoid hemorrhage: prospective cohort study. BMJ. 350:h568. 10.1136/bmj.h568 25694274 PMC4353280

[B38] Perry J. J. Stiell I. G. Sivilotti M. L. Bullard M. J. Emond M. Symington C.. (2011). Sensitivity of computed tomography performed within six hours of onset of headache for diagnosis of subarachnoid haemorrhage: prospective cohort study. BMJ 343:D4277. 10.1136/bmj.d4277 21768192 PMC3138338

[B39] Polet J. D. Weinstein H. C. (1999). Lyme borreliosis and intracranial aneurysm. J. Neurol. Neurosurg. Psychiatry 66, 806–807. 10.1136/jnnp.66.6.806a 10400516 PMC1736382

[B40] Raskind R. (1965). An intracranial arterial aneurysm associated with a recurrent meningioma. J. Neurosurg. 27, 622–625. 10.3171/jns.1965.23.6.0622 5861146

[B41] Raya A. Zipfel G. J. Diringer M. N. Dacey R. G. Jr. Derdeyn C. P. Rich K. M. (2014). Pattern not volume of bleeding predicts angiographic vasospasm in non-aneurysmal subarachnoid hemorrhage. Stroke 45, 265–267. 10.1161/STROKEAHA.113.002629 24193803

[B42] Rinkel G. J. Wijdicks E. F. Hasan D. Kienstra G. E. Franke C. L. Hageman L. M.. (1991a). Outcome in patients with subarachnoid haemorrhage and negative angiography accprding to pattern of haemorrhage on computed tomorgrphy. Lancet 338, 964–968. 10.1016/0140-6736(91)91836-J 1681340

[B43] Rinkel G. J. Wijdicks E. F. Vermeulen M. Ramos L. M. Tanghe H. L. Hasan D.. (1991b). Nonaneurysmal perimesencephalic subarachnoid hemorrhage: CT and MR patterns that differ from aneurysmal rupture. AJNR Am. J. Neuroradiol. 12, 829–834. 1950905 PMC8333493

[B44] Russel C. K. Kershman J. (1937). Spontaneous subarachnoid haemorrhage and brain tumour: (a report of 3 cases). Can. Med. Assoc. 36, 568–577. 20320633 PMC1562212

[B45] Sacco S. Totaro R. Toni D. Marini C. Cerone D. Carolei A. (2009). Incidence, case-fatalities and 10-year survival of subarachnoid hemorrhage in apopulation-based registry. Eur. Neurol. 62, 155–160. 10.1159/000226617 19571544

[B46] Sangwoo Ha Jaeho K. Chong-gue K Suk J. J. (2016). Multiple intracranial aneurysms associated with behçet's disease. J. Cerebrovasc. Endovasc. Neurosurg. 18, 32–37. 10.7461/jcen.2016.18.1.3 27114964 PMC4842906

[B47] Serrone J. C. Tackla R. D. Gozal Y. M. Hanseman D. J. Gogela S. L. Vuong S. M.. (2016). Aneurysm growth and *de novo* aneurysms during aneurysm surveillance. J. Neurosurg. 125, 1374–1382. 10.3171/2015.12.JNS151552 26967775

[B48] Shea A. M. Reed S. D. Curtis L. H. Alexander M. J. Villani J. J. Schulman K. A. (2007). Characteristics of nontraumatic subarachnoid hemorrhage in the United States in 2003. Neurosurgery 61, 1131–1137. 10.1227/01.neu.0000306090.30517.ae 18162891

[B49] Sterne J. A. Hernán M. A. Reeves B. C. Savović J. Berkman N. D. Viswanathan M.. (2016). ROBINS-I: a tool for assessing risk of bias in non randomised studies of interventions. BMJ 355:i4949. 10.1136/bmj.i4919 27733354 PMC5062054

[B50] Suarez J. I. Tarr R. W. Selman W. R. (2006).Aneurysmal subarachnoid hemorrhage. N. Engl. J. Med. 354, 387–396. 10.1056/NEJMra052732 16436770

[B51] Symonds C. P. (1924). Spontaneous subarachnoid haemorrhage. QJM. Os-18, 93–122. 10.1093/qjmed/os-118.69.93

[B52] Trivedi R. A. Watts C. Kirkpatrick P. J. Gillard J. H. (2002). Multiple cerebral aneurysms and the Diamond-Blackfan syndrome. J. Neurol. Neurosurg. Psychiatry. 72, 678–679. 10.1136/jnnp.72.5.678 11971069 PMC1737861

[B53] Van Munster C. E. von und zu Fraunberg M. Rinkel G. J. Rinne J. Koivisto T. Ronkainen A. (2008). Differences in aneurysm and patient characteristics between cohorts of Finnish and Dutch patients with subarachnoid hemorrhage: time trends between 1986 and 2005. Stroke 39, 3166–3171. 10.1161/STROKEAHA.108.516948 18974379

[B54] Vicari P. Choairy A. C. Siufi G. C. Arantes A. M. Fonseca J. R Figueiredo M. S. (2004). Embolization of intracranial aneurysms and sickle cell disease. Am. J. Hematol. 76, 83–84. 10.1002/ajh.20033 15114605

[B55] Vogel T. D. Kulwin C. G. DeNardo A. J. Payner T. D. Boaz J. C. Fulkerson D. H. (2011).Tumor bleeding from a de novo aneurysm associated with optic glioma. J. Neurosurg. Pediatr. 7, 633–636. 10.3171/2011.3.PEDS10562 21631201

[B56] Whiting P. Rutjes A. W. Reitsma J. B. Bossuyt P. M. Kleijnen J. (2013). The development of QUADAS: a tool for the quality assessment of studies of diagnostic accuracy included in systematic reviews. BMC Med. Res. Methodol. 3:25. 10.1186/1471-2288-3-25 14606960 PMC305345

[B57] Whiting P. Savovic J. Higgins J. P. Caldwell D. M. Reeves B. C. Shea B.. (2016). ROBIS: a new tool to assess risk of bias in systematic reviews was developed. J. Clin. Epidemiolog. 69, 225–234. 10.1016/j.jclinepi.2015.06.005 26092286 PMC4687950

[B58] Wilks S. (1859). Guy's Hosp. Rep. 3rd Series 119. Houston, MA: Baylor University Medical Center.

