# Supplementary material for: Novel Subgroups in Subarachnoid Hemorrhage and Their Association With Outcomes—A Systematic Review and Meta-Regression
Source: Front Aging Neurosci. 2021 Jan 11;12:573454. doi: 10.3389/fnagi.2020.573454 (PMC7829354; doi:10.3389/fnagi.2020.573454)
Supplement: Supplementary file 1 [file Data_Sheet_1.docx]

**Novel Subgroups in Subarachnoid Hemorrhage and Their Association With Outcomes– A Systematic Review and Meta-Regression**

*Wang, et al*

Supplementary Appendix-total-1

**Sections page**

**1) Systematic reviews and meta-analyses (PRISMA) guidelines and checklist 1**

**2) The terms used in the search strategy and electronic databases 6**

**3) Parameters of random grouping and definition of parameter name 8**

**4) Risk of Bias in Studies of Interventions (ROBINS-** I**)**  **9**

**5) The 9 risk factors with clear definitions after screening 10**

**6) Statistical analysis -** **(specify which package was used for the analyses and include 13**

**the codes, some additional notes on statistics)**

**7) Stratification SAH related -risk factors 15**

**8) Study selection to be including in the meta-regression were clearly 16**

**Section 1) Supplementary Figure A 1: Flow diagram of study selection in the study**


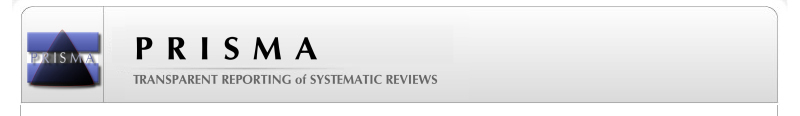
PRISMA 2009 Flow Diagram

Full-text articles excluded, with reasons, Based on full text review:56

aSAH51, naSAH5

Full-text articles assessed for eligibility:989

Records screened:27447

Studies included in qualitative synthesis:933

## Identification

## Eligibility

## Included

## Screening

Article identified through database searching:27447

Article excluded based on title and abstract, DSA, CTA, MRA, CT.

Conference abstract, Animal-based, model, experimental.

Full-text unavailable.

Poor imaging quality.

Duplicated article.

Data, studies without sufficient data for analysis.

removed :26458

aSAH:22793, naSAH:3665

Total studies include in meta-analysis:933

**Section 1) Supplementary Table B 1: Checklist of study selection in the study**

| **Section/topic** | **#** | **Checklist item** | **Reported on page #** |
| --- | --- | --- | --- |
| **TITLE** | | |  |
| Title | 1 | Identify the report as a systematic review, meta-analysis, or both. | Page1 |
| **ABSTRACT** | | |  |
| Structured summary | 2 | Provide a structured summary including, as applicable: background; objectives; data sources; study eligibility criteria, participants, and interventions; study appraisal and synthesis methods; results; limitations; conclusions and implications of key findings; systematic review registration number. | Page1 |
| **INTRODUCTION** | | |  |
| Rationale | 3 | Describe the rationale for the review in the context of what is already known. | Page4 |
| Objectives | 4 | Provide an explicit statement of questions being addressed with reference to participants, interventions, comparisons, outcomes, and study design (PICOS). | Page4 |
| **METHODS** | | |  |
| Protocol and registration | 5 | Indicate if a review protocol exists, if and where it can be accessed (e.g., Web address), and, if available, provide registration information including registration number. | Page5 |
| Eligibility criteria | 6 | Specify study characteristics (e.g., PICOS, length of follow-up) and report characteristics (e.g., years considered, language, publication status) used as criteria for eligibility, giving rationale. | Page6 |
| Information sources | 7 | Describe all information sources (e.g., databases with dates of coverage, contact with study authors to identify additional studies) in the search and date last searched. | Page6 |
| Search | 8 | Present full electronic search strategy for at least one database, including any limits used, such that it could be repeated. | Page6 |
| Study selection | 9 | State the process for selecting studies (i.e., screening, eligibility, included in systematic review, and, if applicable, included in the meta-analysis). | Page6 |
| Data collection process | 10 | Describe method of data extraction from reports (e.g., piloted forms, independently, in duplicate) and any processes for obtaining and confirming data from investigators. | Page7 |
| Data items | 11 | List and define all variables for which data were sought (e.g., PICOS, funding sources) and any assumptions and simplifications made. | Page7 |
| Risk of bias in individual studies | 12 | Describe methods used for assessing risk of bias of individual studies (including specification of whether this was done at the study or outcome level), and how this information is to be used in any data synthesis. | Page8 |
| Summary measures | 13 | State the principal summary measures (e.g., risk ratio, difference in means). | Page8 |
| Synthesis of results | 14 | Describe the methods of handling data and combining results of studies, if done, including measures of consistency (e.g., I^2^) for each meta-analysis. | Page8 |

| **Section/topic** | **#** | **Checklist item** | **Reported on page #** |
| --- | --- | --- | --- |
| Risk of bias across studies | 15 | Specify any assessment of risk of bias that may affect the cumulative evidence (e.g., publication bias, selective reporting within studies). | Page8 |
| Additional analyses | 16 | Describe methods of additional analyses (e.g., sensitivity or subgroup analyses, meta-regression), if done, indicating which were pre-specified. | Page8 |
| **RESULTS** | | |  |
| Study selection | 17 | Give numbers of studies screened, assessed for eligibility, and included in the review, with reasons for exclusions at each stage, ideally with a flow diagram. | Page9 |
| Study characteristics | 18 | For each study, present characteristics for which data were extracted (e.g., study size, PICOS, follow-up period) and provide the citations. | Page9 |
| Risk of bias within studies | 19 | Present data on risk of bias of each study and, if available, any outcome level assessment (see item 12). | Page10 |
| Results of individual studies | 20 | For all outcomes considered (benefits or harms), present, for each study: (a) simple summary data for each intervention group (b) effect estimates and confidence intervals, ideally with a forest plot. | Page10 |
| Synthesis of results | 21 | Present results of each meta-analysis done, including confidence intervals and measures of consistency. | Page11 |
| Risk of bias across studies | 22 | Present results of any assessment of risk of bias across studies (see Item 15). | Page12 |
| Additional analysis | 23 | Give results of additional analyses, if done (e.g., sensitivity or subgroup analyses, meta-regression [see Item 16]). | Page13 |
| **DISCUSSION** | | |  |
| Summary of evidence | 24 | Summarize the main findings including the strength of evidence for each main outcome; consider their relevance to key groups (e.g., healthcare providers, users, and policy makers). | Page14 |
| Limitations | 25 | Discuss limitations at study and outcome level (e.g., risk of bias), and at review-level (e.g., incomplete retrieval of identified research, reporting bias). | Page16 |
| Conclusions | 26 | Provide a general interpretation of the results in the context of other evidence, and implications for future research. | Page17 |
| **FUNDING** | | |  |
| Funding | 27 | Describe sources of funding for the systematic review and other support (e.g., supply of data); role of funders for the systematic review. | Page17 |

**Section 2) The terms used in the search strategy and electronic databases**

The search for the systematic review was performed using electronic databases including: Cochrane, PubMed, Wiley online Library, Web of Science, Springer Link, and Science Direct.

This study strategy included using a keywords, index terms, search terms include ‘(1)aneurysmal OR angiogram positive OR vascular disorder OR sporadic aneurysms; (2) non-aneurysmal OR non aneurysmal OR perimesencephalic OR angiogram-negative;(3) cerebral OR brain OR intracranial OR cerebral cortex OR cortical OR dura;(4)subarachnoid hemorrhage OR SAH OR subarachnoid haemorrhage OR intracranial hemorrhage; (5) angiography OR DSA OR CTA OR MRA; (6)risk factors, OR vessels lesion OR abnormal blood vessels OR arteriovenous malformations, OR trauma OR traumatic brain injury OR diffuse axonal injury, OR neoplastic lesion OR pituitary apoplexy OR cerebral metastases OR malignant glioma OR acoustic neuroma OR schwannoma of the cranial nerve OR meningiomas OR cervical spinal cord hemangioblastoma OR spinal meningeal carcinomatosis OR glioma OR retinoblastoma OR acoustic neurilemmoma OR rathke’s cleft cysts OR cerebral malformations, OR Inflammatory disease OR Lyme borreliosis OR intracranial mycotic aneurysms OR behçet’s disease OR primary angiitis OR polyarteritis nodosa, OR hematologic disease OR blood disease OR leukemia OR sickle-cell anemia OR diamond-blackfan anemia OR idiopathic thrombocytopenic purpura OR acute lymphoblastic leukemia OR Afibrinogenemia, OR drugs OR toxic OR cocaine OR anti-epileptic drug OR methamphetamine OR heroin OR amphetamine, OR perimesencephalic hemorrhage OR non-idiopathic perimesencephalic hemorrhage, OR non-inflammatory lesion OR carotid artery dissection OR dural arteriovenous fistulas OR cerebral amyloid angiopathy OR cerebral venous thrombosis, OR hypertension OR systemic hypertension, OR atherosclerosis OR total cholesterol OR high-desity lipoproteins OR low-density lipoprotenins. The retrieval formula was as follows (1) AND (2) AND (3) AND (4) AND (5) AND (6).

**The specific related disease**

These risk factors can be categorized into aSAH and naSAH-related. Related SAH presenting disease included: *non-aneurysmal-non-inflammatory vascular lesions* -carotid artery dissection (CAD), dural arteriovenous fistulas (DAVF), cerebral amyloid angiopathy (CAA), and cerebral venous thrombosis (CVT). *Neoplastic lesion*- pituitary apoplexy(PA), cerebral metastases(CM), malignant glioma(MG), acoustic neuroma(AN), schwannoma of the cranial nerve, meningiomas, cervical spinal cord hemangioblastoma, and spinal meningeal carcinomatosis, glioma, retinoblastoma, acoustic neurilemmoma, Rathke’s cleft cysts (RCC), and cerebral malformations. *Vessels lesion*- Arteriovenous malfmations(AVMs). *Inflammatory lesion*-Lyme borreliosis, intracranial mycotic aneurysms (IMA), Behçet’s disease (BD), primary angiitis, and polyarteritis nodosa (PAN). *Blood disease* (Hematologic disease)- leukemia, Sickle-cell anemia (SCA), Diamond-Blackfan anemia (DBA), idiopathic thrombocytopenic purpura (ITP), acute lymphoblastic leukemia (ALL), Afibrinogenemia. *traumatic* brain injury (TBI). Diffuse axonal injury (DAI). *Idiopathic* perimesencephalic hemorrhage (IPH), non-Idiopathic perimesencephalic hemorrhage.

**Section 3) Parameters of random grouping and definition of parameter name (Split the dataset into training, test and validation sets)**

Enrolled in diagnosed SAH patients

aSAH (n=33373)

naSAH (n=9778)

Candidate novel related SAH risk factors discovery

Differential risk factors assessed

Integrative differ factors: aSAH and naSAH analysis

Survival analysis for individual risk factors

N=43151

Discovery cohort

**Model**

**Validation**

**Model**

**Training，Test**

validation1 validation 2

aSAH naSAH

(n=10012) (n=2933)

Training Cohort Test Cohort

(aSAH: n=16687) ( aSAH: n=6674)

(na-SAH:n=4889) (na-SAH:n=1956)

Training and Test Cohort

Schematic diagram of our experimental design and possible outcome trajectories of patients with SAH. Samples were consisted of two batches: the discovery cohort consisted aSAH of diagnostic samples(n=33373), but also included naSAH(n=9778); train and test cohort consisted of patients from the aSAH and naSAH patients (n=16687, 4889) (n=6774, 1956). validation cohort consisted of patients from the aSAH and naSAH patients (n=10012, 2933). study design for the training, test and validation of patients with SAH.

we divided the discovery cohort training and test cohorts, which consisted of two thirds and one third, respectively, of patients in the discovery cohort ^1^ .

**Section 4)**  **Risk of Bias in Non-randomised Studies of Interventions (ROBINS-** I**)**

This tool evaluates the risk of bias in non-randomised studies and randomized controlled study in seven domains: (1) bias due to confounding; (2) bias in participant selection; (3) bias in classification of interventions; (4) bias due to deviations from intended interventions; (5) bias due to missing data; (6) bias in measurement of outcomes; (7) bias in selection of reported results. Each domain will be classified as either low, high or unclear risk of bias ^2^. This is a standard tool to detect any bias such as selection bias, performance bias, detection bias, attrition bias, reporting and other bias.

Using the ROBIS tool, each study judged on seven items: random sequence generation (selection bias), allocation concealment (selection bias), blinding of participants and personnel (performance bias), blinding of outcome assessment (detection bias), incomplete outcome data (attrition bias), selective reporting (reporting bias) and other relevant potential bias (cross over). The tool is completed in four phases: identification and selection phase, data collection and study appraisal phase, synthesis and finding phase, risk of bias in the review phase. Review Manager software, version 5.0 (the Nordic Cochrane Centre, Copenhagen, Denmark) was used to determine the risk of bias in differ studies.

**Section 5)**  **The 9 risk factors with clear definitions after screening**

*Aneurysmal-blood disease subarachnoid haemorrhage* (a-bd-SAH): refers to the patients with blood disease include Leukaemia, hemophilia, sickle cell anaemia, pernicious anaemia, aplastic anaemia, agranulocytosis, thrombocytopenic purpura, polycythaemia vera, Waldenström’s macroglobulinaemia, lymphoma, myeloma, hereditary spherocytosis, afibrinogenaemia, liver diseases associated with coagulopathy, disseminated intravascular coagulation, acquired coagulopathies due to anticoagulant drugs, other congenital or acquired platelet vessel or coagulation disorders related SAH have an aneurysm. CT scans show the pattern of hemorrhage in the superficial cortical sulci; angiograms reveal intracranial aneurysm (multiple distal branch occlusion and leptomeningeal collateral circulation) ^3,4^.

*Aneurysmal-drugs subarachnoid haemorrhage* (a-d-SAH): (Drug/Toxins) refers to the patients with amphetamines, cocaine abuse, morphine related SAH have an aneurysm. CT scans show the pattern of SAH. angiograms reveal intracranial aneurysm ^5,6^.

*Aneurysmal Subarachnoid hemorrhage co-existing with Inflammatory lesion* (a-SAH-I): refers to the patients with Lyme borreliosis, intracranial mycotic aneurysms (IMA), Behçet’s disease (BD), primary angiitis, and polyarteritis nodosa (PAN), bacterial, tuberculous, and fungal meningitis, malaria, mycotic infective endocarditis and aspergillosis related SAH have an aneurysm. CT scans show pattern of hemorrhage indistinguishable from saccular aneurysm; the intracranial aneurysm found on angiograms ^7,8^.

*Aneurysmal Subarachnoid hemorrhage co-existing neoplastic (*a-SAH-N); refers to the patients with pituitary apoplexy, cerebral metastases, malignant glioma, acoustic neuroma, schwannoma of the cranial nerve, meningiomas, cervical spinal cord hemangioblastoma, and spinal meningeal carcinomatosis, glioma, retinoblastoma, acoustic neurilemmoma, Rathke’s cleft cysts (RCC), and cerebral malformations related SAH have an aneurysm. CT scans show the pattern of SAH. angiograms reveal intracranial aneurysm ^9,10^.

*Aneurysmal Subarachnoid hemorrhage-simple*(a-SAH-S); refers to the patients with rupture of an intracranial aneurysm alone causes subarachnoid haemorrhage and influenced by the presence of positive family history, age, and sex; but not influenced by the presence of blood disease, Drug/Toxins, Inflammatory lesion, system/cerebral neoplastic, tramua, and vesselas lesion(When an aneurysm was found, there was no other systemic disease). Angiograms reveal aneurysm, of these include unruptured intracranial aneurysm (UIAs) ^11^.

*Aneurysmal Subarachnoid hemorrhage co-existing trauma* (a-SAH-T); refers to this type of patients with traumatic brain injury (TBI) related SAH have an aneurysm. CT scans show the pattern of SAH. angiograms reveal intracranial aneurysm ^12^.

*Aneurysmal Subarachnoid hemorrhage co-existing vesselas lesion* (a-SAH-V); refers to the patients with cerebral arterio-venous malformation (AVMs) related SAH have an aneurysm. CT scans show the pattern of SAH; Digital subtraction angiography (DSA) should be performed and determine there is a nidal or perinidal aneurysm in every patient^13^.

*Non-aneurysmal-drugs subarachnoid haemorrhage* (na-d-SAH): refers to this type of patients with amphetamines, cocaine abuse, morphine related SAH have a normal catheter cerebral angiography, no source of haemorrhage evident on other neuroimaging studies. CT scans show the pattern of SAH ^14^.

*Non-aneurysmal-neoplastic subarachnoid haemorrhage* (na-ne-SAH) : refers to this type of patients with pituitary apoplexy, cerebral metastases, malignant glioma, acoustic neuroma, schwannoma of the cranial nerve, meningiomas, cervical spinal cord hemangioblastoma, and spinal meningeal carcinomatosis, glioma, retinoblastoma, acoustic neurilemmoma, Rathke’s cleft cysts related SAH have a normal catheter cerebral angiography. CT scans show the pattern of SAH; no source of haemorrhage evident on other neuroimaging studies ^15^.

*Non-aneurysmal& non- inflammatory &intracerebral vascular lesion subarachnoid haemorrhage* (na-ni-ivl-SAH): refers to this type of patients with Carotid artery dissection (CAD), Dural arteriovenous fistulas (DAVF), Cerebral amyloid angiopathy (CAA) related SHA have a normal catheter cerebral angiography; CT scans predominantly show cortical sulci and convex pattern of bleeding. with negative angiography for aneurysm ^16^.

*Non-aneurysmal-traumatic subarachnoid haemorrhage (*na-t-SAH): refers to this type of patients with traumatic brain injury (TBI) related SAH have not found an aneurysm. Nonenhanced CT obtained after sustaining the head injury, and scans show the pattern of SAH. angiograms reveal was negative^17^.

*Non-aneurysmal perimesencephalic SAH* (na-pmSAH): refers to this type of patients can occur in any patients over the age of 20 to sixth decade. CT scans show Centre of the bleeding located immediately anterior to the midbrain, with or without extension of blood to the anterior part of the ambient cistern or to the basal part of the sylvian fissure; no extension to the anterior interhemispheric fissure and no extension to the lateral sylvian fissue, except for a small amount of blood; and absence of frank intraventricular haemorrhage^18^. Angiograms no reveal aneurysm. Good outcome and absence of rebleeding in long term.

**Section 6) Detailed statistical methods**

The different statistical methods used in each part of the section are detailed below.

Statistical analysis

R code

m1<- read.csv(file.choose())

train <-createDataPartition(y=m1$Creditability, p=0.75,list=FALSE)

train2 <- m1[train, ]

test2 <- m1[-train, ]

library(metafor)

library(meta)

rate<-transform (m1, p= case/sample.size)

shapiro.test(rate$p)

meta1 <- metaprop (sample.size,case,data=m1, studlab=paste(Study.ID),sm="PRAW")

forest(meta1)

metabias(meta1, method.bias="peters")

funnel(meta1)

metabias(meta1, method="linreg")

tf1 <- trimfill(meta1, comb.fixed=TRUE)

summary(tf1)

funnel(tf1)

meta1 <- metaprop (case,sample.size, data=m1, studlab=paste(Study.ID),sm="PAS")

data1<-metabin(event,n,data=data1,sm="OR",

studlab=paste(Study.ID,year),comb.random=FALSE)

metabias(data1, method.bias="peters")

funnel(m1)

R code divided into Training, Test, Validation

m1<- read.csv(file.choose())

train.rows <- sample(rownames(m1), dim(m1)[1]*0.5)

valid.rows <- sample(setdiff(rownames(m1), train.rows), dim(m1)[1]*0.3)

test.rows <- setdiff(rownames(m1), union(train.rows, valid.rows))

m1.train <- m1 [train.rows, ]

m1.valid <- m1 [valid.rows, ]

m1.test <- m1[test.rows, ]

**Some additional notes on statistics**

*Individual risk factors discovery and analyses-*aSAH and naSAH-related risk factors discovery and analyses

For 11 association of risk factors (drugs, trauma, neoplastic, vessels lesion, Inflammatory lesion, blood disease, aneurysm, perimsencephalic haemorrhage, Hypertension, ﻿Atherosclerosis, Congenital disease (NF-1, Marfan syndrome)), selected bases solely on availability of cerebrovascular accident or stroke. The combined OR is calculated as Mantel-Haenszel weighted average of OR. Since the real therapeutic effects of various post-processing schemes may differ in the include trials, so random-effects models used in the analysis. A total of 933 article were identified (933 studies form random forest algorithm and univariate Cox regression analysis). Receiver operating characteristic curve (ROC) and the area under the curve (AUC) were used to analyze survival outcome of patients.

*Identification of optimal risk factors and subtypes of SAH*

SAH can categorized into two type, which may be in either aneurysmal SAH or non-aneurysmal SAH, predominantly diagnosis in digital subtract angiography (DSA) of the whole cerebral vessels will shows whether or not the presence of aneurysms. we will review SAH type in clinical practice and research to identification of optimal risk factors. aSAH-S versus a-d-SAH, aSAH-S versus aSAH-T, aSAH-S versus aSAH-N, aSAH-S versus aSAH-V, aSAH-S versus aSAH-I, aSAH-S versus a-bd-SAH. na-pmSAH, na-ni-ivl-SAH, na-t-SAH, na-ne-SAH, na-d-SAH.

*Risk factor of SAH progression*

Follow-up and survival functions (survival ratio with its corresponding 95% confidence interval (CI)) were estimated by the Kaplan Meier survival curves method and evaluated compared using a log-rank test.

**Section 7) Stratification SAH related -risk factors**

The factor, which make the patients more prone to develop SAH, have to be considered in view of a cerebrovascular disease risk profile, which usually contains between blood disease, inflammatory disease, non-inflammatory vascular lesions, neoplastic lesion, trauma, Vessels lesion, and drug. Furthermore, presence of simple aneurysm and perimsencephalic haemorrhage were added to the risk profile of cerebrovascular disease.

The most frequent risk factor in subgroup with a-SAH and subgroup naSAH was demonstrated significant differences between clinical group. Pairwise comparisons (Dunn test with Bonferoni correction) showed ① for aSAH-S versus a-d-SAH, aSAH-S versus aSAH-T, aSAH-S versus aSAH-N, aSAH-S versus aSAH-V, aSAH-S versus aSAH-I, aSAH-S versus a-bd-SAH. ② for na-pmSAH versus na-ni-ivl-SAH, na-pmSAH versus na-t-SAH, na-pmSAH versus na-ne-SAH, na-pmSAH versus na-d-SAH(Table.2).

**Section 8) Study selection to be including in the meta-regression were clearly and a few notes on inclusion literature**

With the risk factors that cause and aggressive bleeding are mixed in different types of literature, of these include case reports and case series, it is necessary and appropriate to include all studies in the meta-analysis. Considerable evidence implicates case report and case series are related to SAH subtype. Such as cocaine abuse related to SAH.

REFERENCE

1. Emilia L. Lim, Diane L. Trinh, Rhonda E. Ries, Jim Wang, Robert B. Gerbing, Yussanne Ma, James Topham, Maya Hughes, Erin Pleasance, Andrew J. Mungall, Richard Moore, Yongjun Zhao, Richard Aplenc, Lillian Sung, E. Anders Kolb, Alan Gamis, Malcolm Smith, Daniela S. Gerhard, Todd A. Alonzo, Soheil Meshinchi, and Marco A. Marra. MicroRNA Expression-Based Model Indicates Event-Free Survival in Pediatric Acute Myeloid Leukemia. J Clin Oncol. 2017; 35:3964-3977
2. Higgins JP, Altman DG, Gøtzsche PC, et al. The Cochrane Collaboration’s tool for assessing risk of bias in randomised trials.BMJ. 2011;343: d5928.
3. Carey J, Numaguchi Y, Nadell J. Subarachnoid hemorrhage in sickle cell disease. Childs Nerv Syst. 1990; 6:47-50.
4. R Loch Macdonald, Tom A Schweizer. Spontaneous subarachnoid haemorrhage. Lancet 2017; 389: 655–66
5. J. M. Lappin, S. Darke, and M. Farrell, “Stroke and methamphetamine use in young adults: a review,” Journal of Neurology, Neurosurgery & Psychiatry, 2017; 88(12): 1079–1091.
6. Kalani R, Liotta EM, Prabhakaran S. [Diagnostic Yield of Universal Urine Toxicology Screening in an Unselected Cohort of Stroke Patients.](https://pubmed.ncbi.nlm.nih.gov/26675665/?from_term=drug%2C+cocaine+use+SAH&from_pos=6) PLoS One. 2015;10(12): e0144772.
7. [Robert D Brown Jr](https://pubmed.ncbi.nlm.nih.gov/?term=Brown+RD+Jr&cauthor_id=24646873) , [Joseph P Broderick](https://pubmed.ncbi.nlm.nih.gov/?term=Broderick+JP&cauthor_id=24646873) . Unruptured Intracranial Aneurysms: Epidemiology, Natural History, Management Options, and Familial Screening. Lancet Neurol. 2014;13(4):393-404
8. Zanaty M, Chalouhi N, Starke RM, Tjoumakaris S, Gonzalez LF, Hasan D, Rosenwasser R, Jabbour P. [Endovascular treatment of cerebral mycotic aneurysm: a review of the literature and single center experience.](https://pubmed.ncbi.nlm.nih.gov/24383049/?from_term=mycotic+infective+%2C+Intracranial+aneurysm&from_page=3&from_pos=1) Biomed Res Int. 2013;2013:151643.
9. Pant B, Arita K, Kurisu K, Tominaga A, Eguchi K, Uozumi T. [Incidence of intracranial aneurysm associated with pituitary adenoma.](https://pubmed.ncbi.nlm.nih.gov/9085282/?from_term=pituitary+adenoma+%2C+Intracranial+aneurysm&from_pos=4) Neurosurg Rev. 1997;20(1):13-7.
10. Zheng J, Zhang J. [Neoplastic cerebral aneurysm from metastatic tumor: a systematic review of clinical and treatment characteristics.](https://pubmed.ncbi.nlm.nih.gov/25484302/?from_term=cerebral+metastases%2C+Intracranial+aneurysm&from_pos=1) Clin Neurol Neurosurg. 2015;128:107-11.
11. Brown RD Jr, Broderick JP. [Unruptured intracranial aneurysms: epidemiology, natural history, management options, and familial screening.](https://pubmed.ncbi.nlm.nih.gov/24646873/?from_term=+Intracranial+aneurysm&from_pos=1) Lancet Neurol. 2014;13(4):393-404.
12. Haddad FS, Haddad GF, Taha J. [Traumatic intracranial aneurysms caused by missiles: their presentation and management.](https://pubmed.ncbi.nlm.nih.gov/1994264/) Neurosurgery. 1991;28(1):1-7.
13. Jha V, Behari S, Jaiswal AK, Bhaisora KS, Shende YP, Phadke RV. [The "focus on aneurysm" principle: Classification and surgical principles of management of concurrent arterial aneurysm with arteriovenous malformation causing intracranial hemorrhage.](https://pubmed.ncbi.nlm.nih.gov/27366251/?from_term=Intracranial+aneurysm%2C+AVM&from_page=2&from_pos=5) Asian J Neurosurg. 2016;11(3):240-54
14. [Bradley A Gross](https://pubmed.ncbi.nlm.nih.gov/?term=Gross+BA&cauthor_id=22588341) , [Ning Lin](https://pubmed.ncbi.nlm.nih.gov/?term=Lin+N&cauthor_id=22588341), [Kai U Frerichs](https://pubmed.ncbi.nlm.nih.gov/?term=Frerichs+KU&cauthor_id=22588341), [Rose Du](https://pubmed.ncbi.nlm.nih.gov/?term=Du+R&cauthor_id=22588341). Vasospasm After Spontaneous Angiographically Negative Subarachnoid Hemorrhage. Acta Neurochir (Wien). 2012;154(7):1127-33
15. [Min-Su Kim](https://pubmed.ncbi.nlm.nih.gov/?term=Kim+MS&cauthor_id=21887396) , [Sang Woo Kim](https://pubmed.ncbi.nlm.nih.gov/?term=Kim+SW&cauthor_id=21887396), [Chul-Hoon Chang](https://pubmed.ncbi.nlm.nih.gov/?term=Chang+CH&cauthor_id=21887396), [Oh-Lyong Kim](https://pubmed.ncbi.nlm.nih.gov/?term=Kim+OL&cauthor_id=21887396). Cerebellar Pilocytic Astrocytomas With Spontaneous Intratumoral Hemorrhage in Adult. J Korean Neurosurg Soc. 2011;49(6):363-6
16. Cooke DL, Stout CE, Kim WT, Kansagra AP, Yu JP, Gu A, Jewell NP, Hetts SW, Higashida RT, Dowd CF, Halbach VV. [Cerebral arterial fenestrations.](https://pubmed.ncbi.nlm.nih.gov/24976087/?from_term=Dural+arteriovenous+fistulas+%2Cnon-aneurysmal&from_pos=1) Interv Neuroradiol. 2014;20(3):261-74
17. Bruder N. [Non-aneurysmal subarachnoid haemorrhage].](https://pubmed.ncbi.nlm.nih.gov/17936566/?from_term=traumatic+brain+injury+%2C+non-aneurysmal&from_pos=2) Ann Fr Anesth Reanim. 2007;26(11):954-8
18. Gabriel J.E.Rinkel, Eelco F.M.Wijdcks, Djo Hasan, Georg E.M.Kienstra, Cees L. Franke, Loes M. Hageman, Marinus Vermeulen, Jan Van Gijn. Outcome in patients with subarachnoid haemorrhage and negative angiography according to pattern of haemorrhage on computed tomorgrphy. The Lancet 1991; 338: 964-968.
