# Supplementary material for: Novel Subgroups in Subarachnoid Hemorrhage and Their Association With Outcomes—A Systematic Review and Meta-Regression
Source: Front Aging Neurosci. 2021 Jan 11;12:573454. doi: 10.3389/fnagi.2020.573454 (PMC7829354; doi:10.3389/fnagi.2020.573454)
Supplement: Supplementary file 2 [file Data_Sheet_2.docx]

**Novel Subgroups in Subarachnoid Hemorrhage and Their Association With Outcomes– A Systematic Review and Meta-Regression**

*Wang, et al*

Supplementary Appendix-2.1

**Sections page**

1. **Supplementary Appendix. Figure.S1-7---** **aSAH subtype 1**

**Forest plot and Funnel plots**


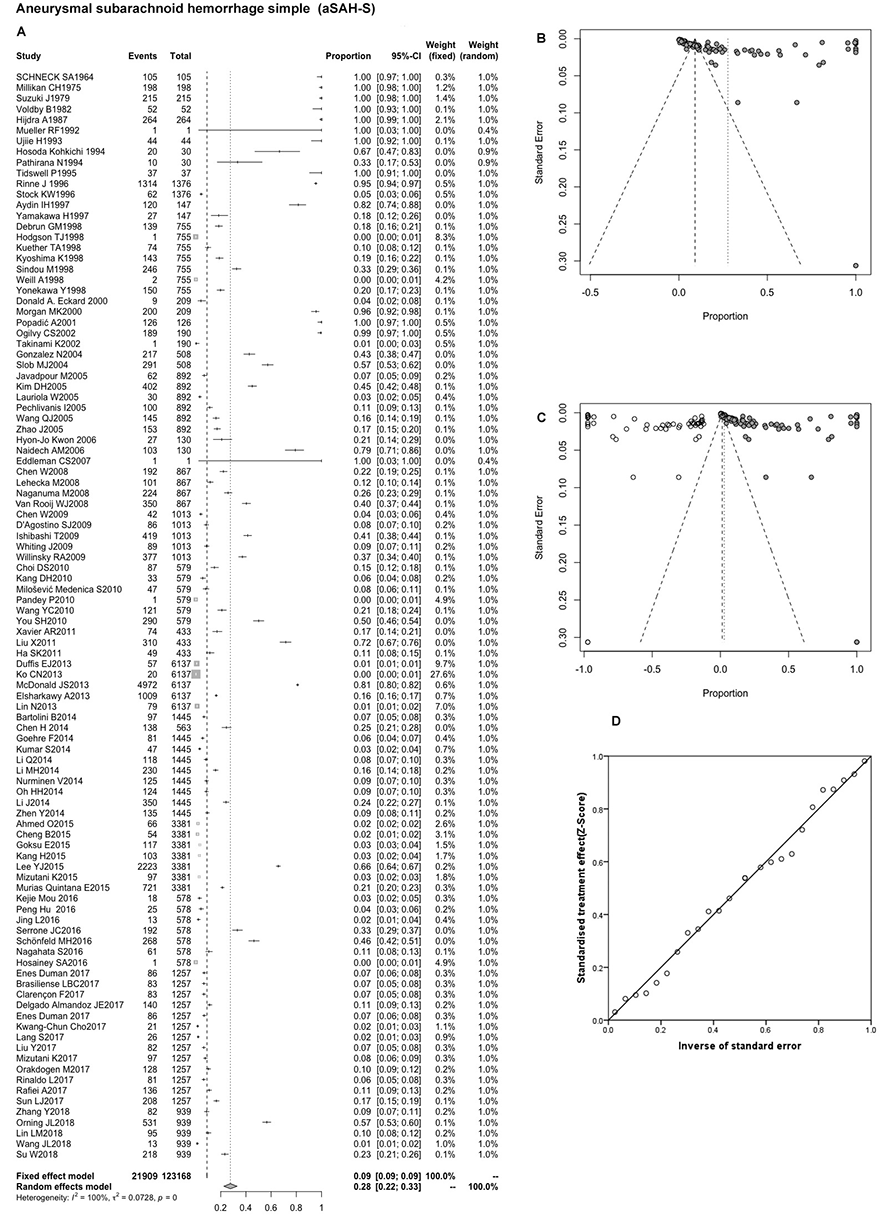


**Figure S1.** **Forest plots for the subgroup analysis of population–based aneurysmal subarachnoid hemorrhage simple (aSAH-S) prevalence.** (A): Forest plot of the subgroup analysis by aSAH-S. (B): Funnel plots of the incidence for all studies in Meta-analysis. each point represents a separate study in the indicated association after 102studies were filled by a nonparametric trim and fill method (the solid circle represented studies which were filled. (C) Trim-and fill funnel plots were used to observe and adjust publication bias and asymmetries. (D): Linear regression test of funnel plot asymmetry (Egger test, Kolmogorov-Smirnov Z test). The intercept indicating Normal distribution is 0.3017(The intercept indicating bias is 17.7583). P-value = 0.9748, indicating significant publication Normal distribution.

**
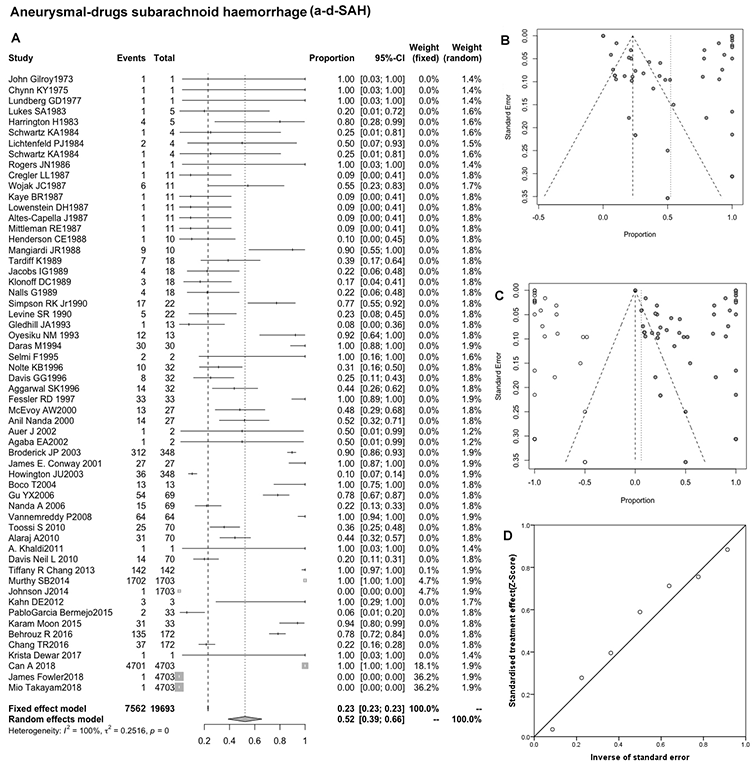
**

**Figure S2.** **Forest plots for the subgroup analysis of population–based aneurysmal drug subarachnoid hemorrhage (a-d-SAH) prevalence.** (A): Forest plot of the subgroup analysis by a-d-SAH. (B): Funnel plots, each point represents a separate study in the indicated association after 58 studies were filled by a nonparametric trim and fill method (the solid circle represented studies which were filled. (C): Trim-and fill funnel plots were used to observe and adjust publication bias and asymmetries. (D): Linear regression test of funnel plot asymmetry (Kolmogorov-Smirnov Z test). The intercept indicating Normal distribution is 0.4562 P-value = 0.5656, indicating significant publication Normal distribution

**
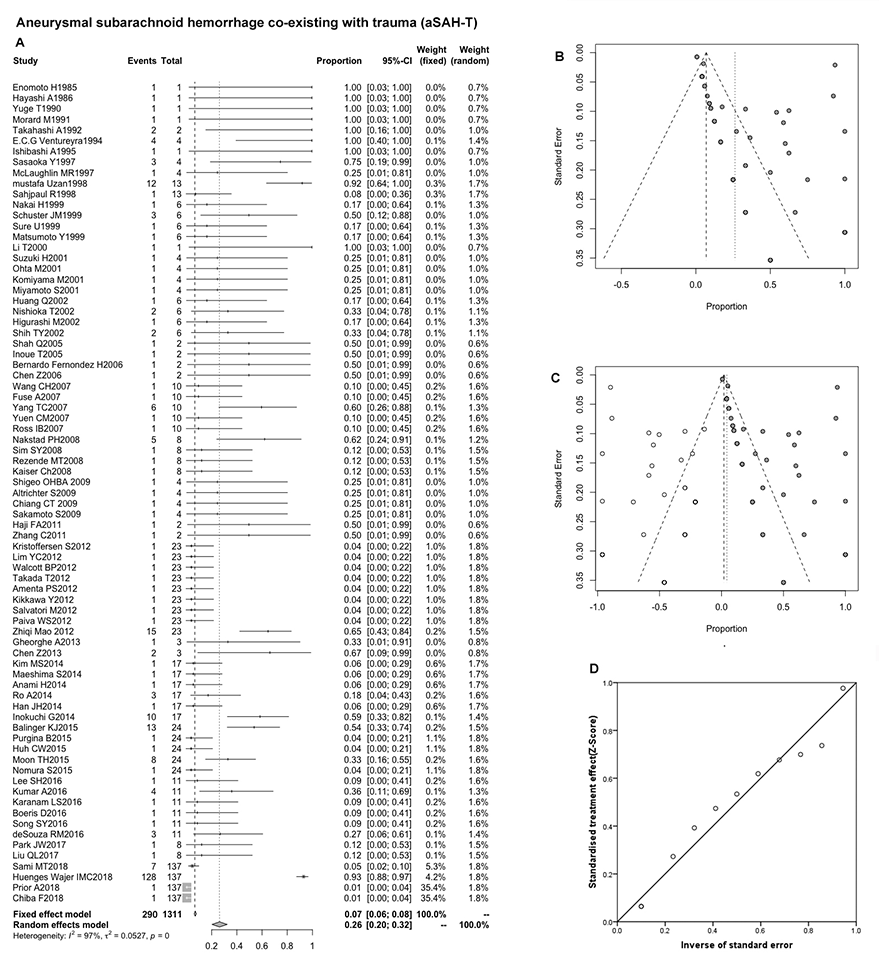
**

**Figure S3.** **Forest plots for the subgroup analysis of population–based aneurysmal subarachnoid hemorrhage coexisting with trauma (aSAH-T) prevalence.** (A): Forest plot of the subgroup analysis by aSAH-T. (B): Funnel plots, each point represents a separate study in the indicated association after77 studies were filled by a nonparametric trim and fill method (the solid circle represented studies which were filled. (C): Trim-and fill funnel plots were used to observe and adjust publication bias and asymmetries. (D): Linear regression test of funnel plot asymmetry (Kolmogorov-Smirnov Z test). The intercept indicating Normal distribution is 0.3450. P-value = 0.9231, indicating significant publication Normal distribution

**
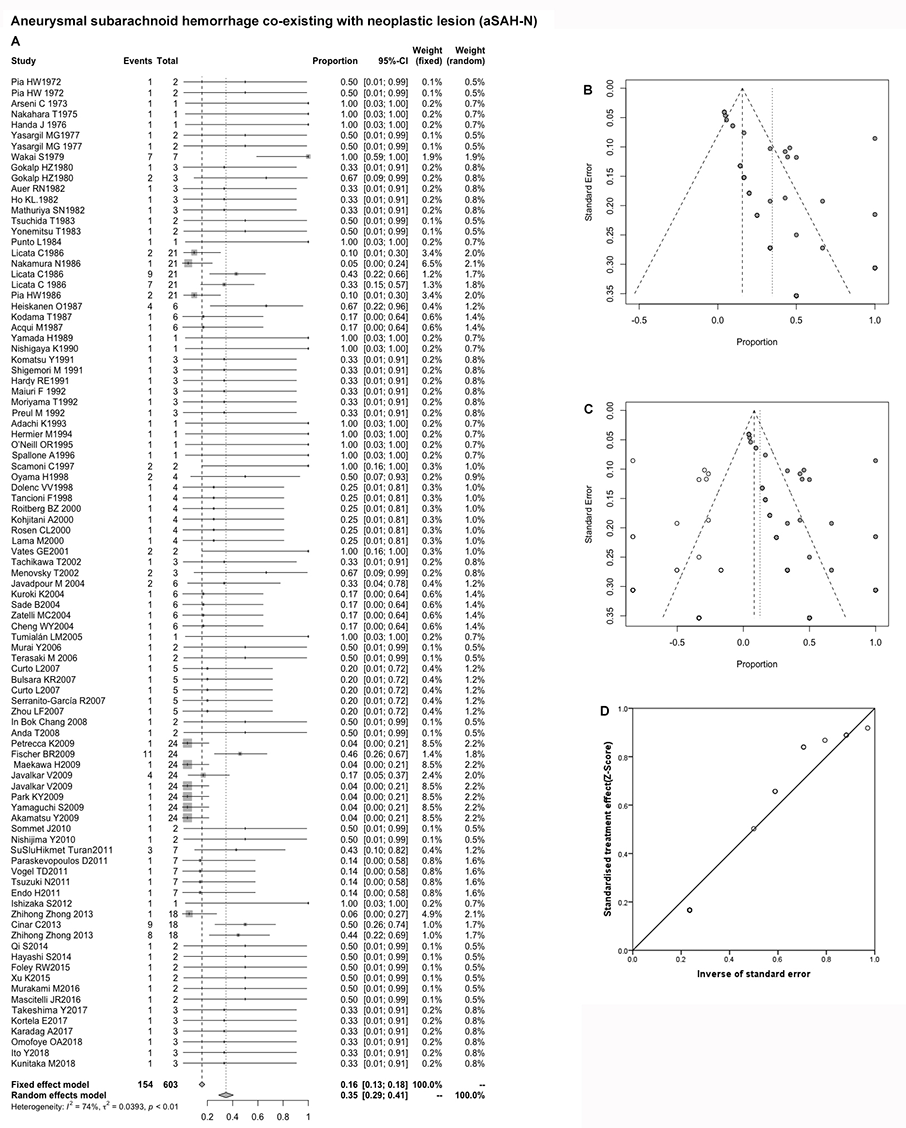
**

**Figure S4.** **Forest plots for the subgroup analysis of population–based aneurysmal subarachnoid hemorrhage coexisting with neoplastic lesion (aSAH-N) prevalence.** (A): Forest plot of the subgroup analysis by aSAH-N. (B): Funnel plots, each point represents a separate study in the indicated association after 89studies were filled by a nonparametric trim and fill method (the solid circle represented studies which were filled. (C):Trim-and fill funnel plots were used to observe and adjust publication bias and asymmetries. (D): Linear regression test of funnel plot asymmetry (Kolmogorov-Smirnov Z test). The intercept indicating Normal distribution is 0.2585. P-value = 0.0007, indicating significant publication Normal distribution

**
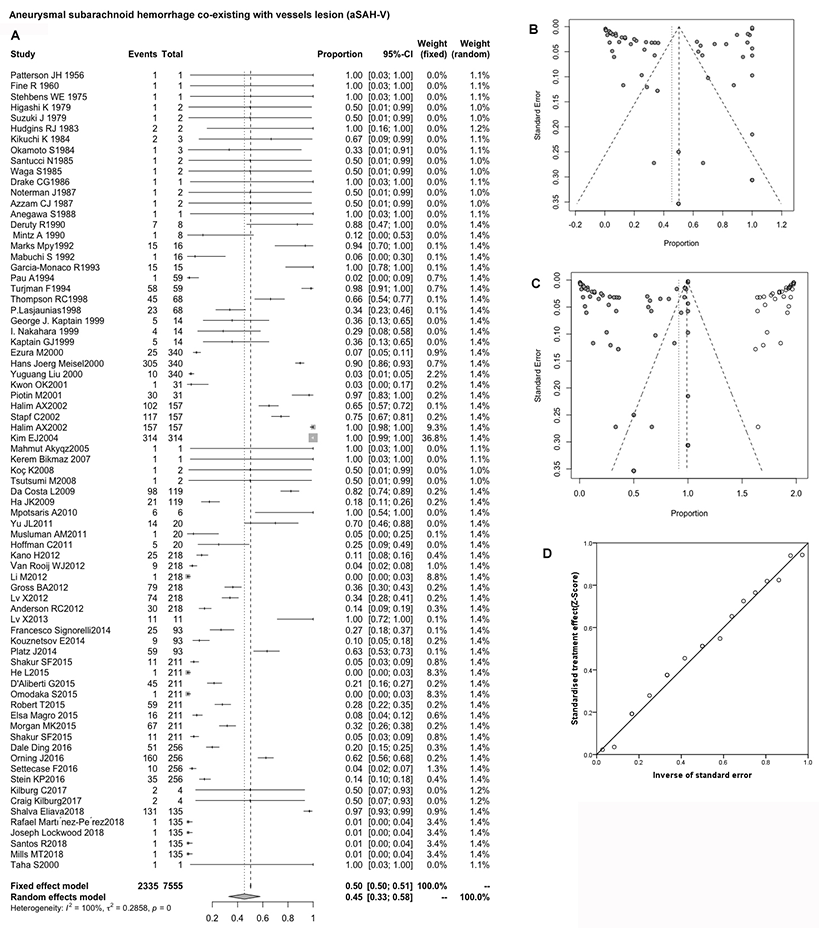
**

**Figure S5**. **Forest plots for the subgroup analysis of population–based aneurysmal subarachnoid hemorrhage coexisting with vessels lesion (aSAH-V) prevalence.** (A): Forest plot of the subgroup analysis by aSAH-V. (B): Funnel plots, each point represents a separate study in the indicated association after 75studies were filled by a nonparametric trim and fill method (the solid circle represented studies which were filled. (C):Trim-and fill funnel plots were used to observe and adjust publication bias and asymmetries. (D): Linear regression test of funnel plot asymmetry (Kolmogorov-Smirnov Z test). The intercept indicating Normal distribution is 0.3635. P-value = 0.56, indicating significant publication Normal distribution

**
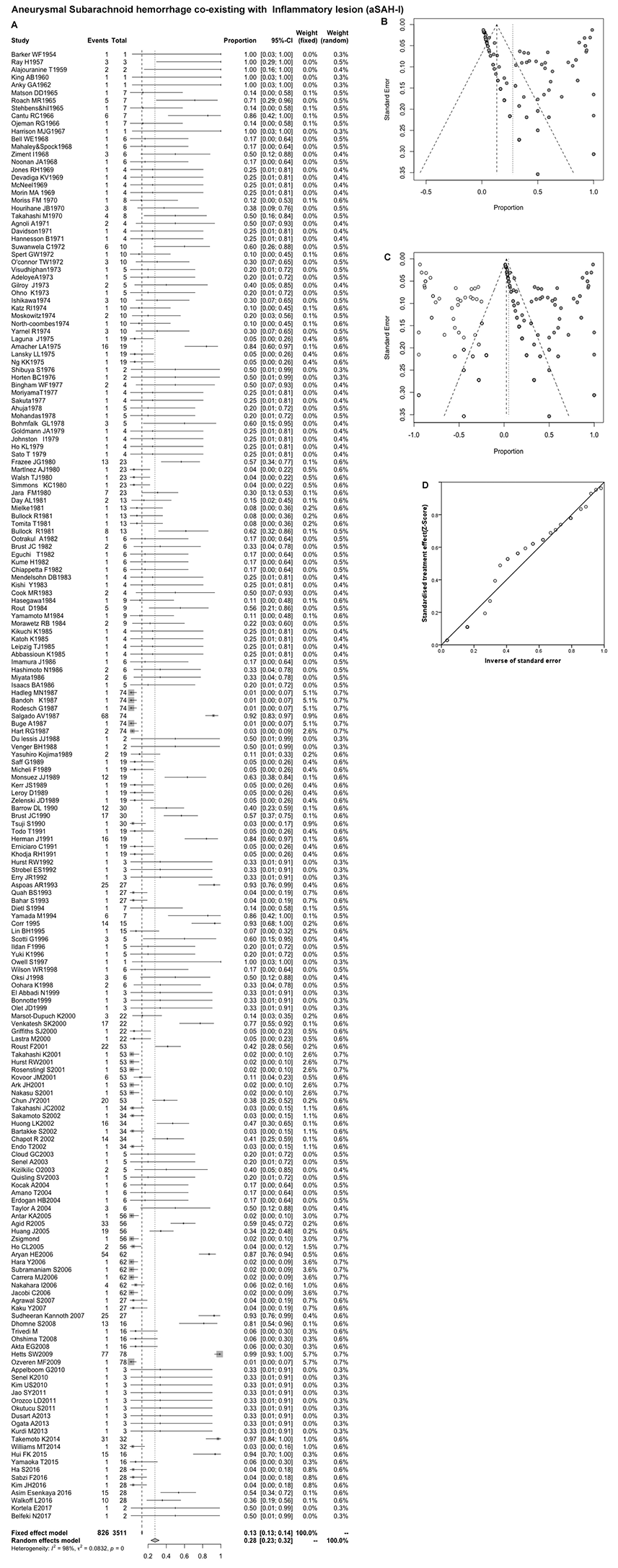
**

**Figure S6**. **Forest plots for the subgroup analysis of population–based aneurysmal subarachnoid hemorrhage coexisting with inflammatory lesion (aSAH-I) prevalence.** (A): Forest plot of the subgroup analysis by aSAH-I. (B): Funnel plots, each point represents a separate study in the indicated association after 191 studies were filled by a nonparametric trim and fill method (the solid circle represented studies which were filled. (C): Trim-and fill funnel plots were used to observe and adjust publication bias and asymmetries. (D): Linear regression test of funnel plot asymmetry (Kolmogorov-Smirnov Z test). The intercept indicating Normal distribution is 0.3854. P-value = 0.2828, indicating significant publication Normal distribution

**
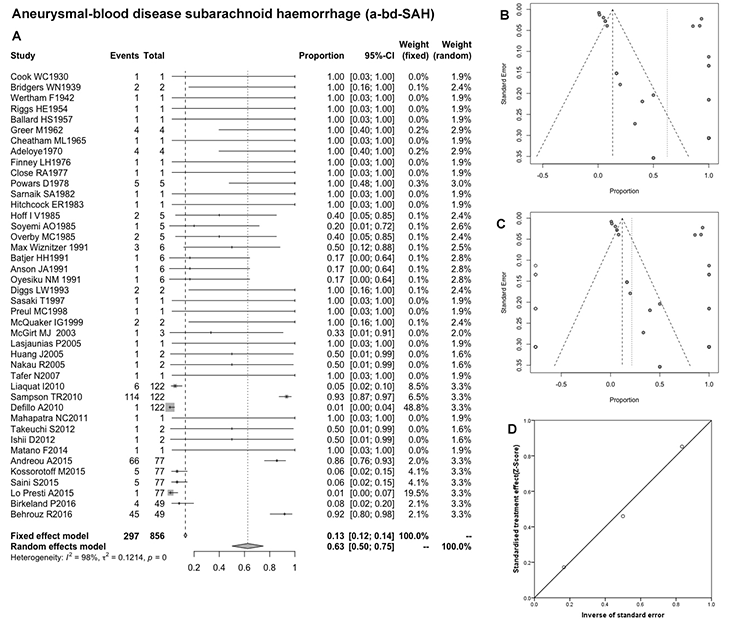
**

**Figure S7**. **Forest plots for the subgroup analysis of population–based aneurysmal blood disease subarachnoid hemorrhage (a-bd-SAH) prevalence.** (A): Forest plot of the subgroup analysis by a-bd-SAH. (B): Funnel plots, each point represents a separate study in the indicated association after 42 studies were filled by a nonparametric trim and fill method (the solid circle represented studies which were filled). (C):Trim-and fill funnel plots were used to observe and adjust publication bias and asymmetries. (D): Linear regression test of funnel plot asymmetry (Kolmogorov-Smirnov Z test). The intercept indicating Normal distribution is 0.4569. P-value = 0.6858, indicating significant publication Normal distribution
