# Supplementary material for: Novel Subgroups in Subarachnoid Hemorrhage and Their Association With Outcomes—A Systematic Review and Meta-Regression
Source: Front Aging Neurosci. 2021 Jan 11;12:573454. doi: 10.3389/fnagi.2020.573454 (PMC7829354; doi:10.3389/fnagi.2020.573454)
Supplement: Supplementary file 3 [file Data_Sheet_3.docx]

**Novel Subgroups in Subarachnoid Hemorrhage and Their Association With Outcomes– A Systematic Review and Meta-Regression**

*Wang, et al*

Supplementary Appendix-2.2

**Sections page**

1. **Supplementary Appendix. Figure. S8-12---** **naSAH subtype 1**

**Forest plot and Funnel plots**

**
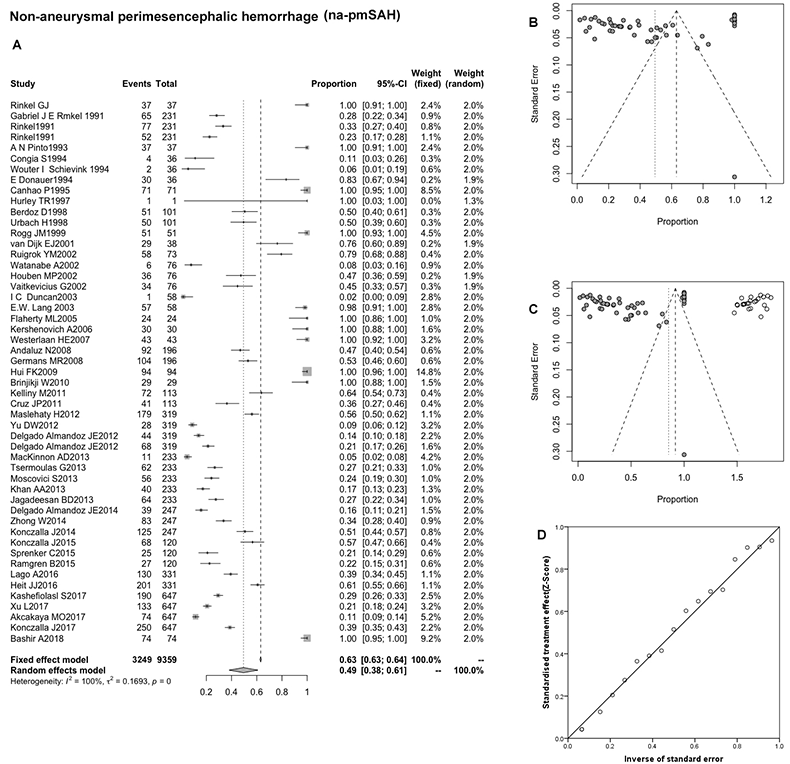
**

**Figure S8.** **Forest plots for the subgroup analysis of population–based non-aneurysmal perimesencephalic subarachnoid hemorrhage(na-pmSAH) prevalence.** (A): Forest plot of the subgroup analysis by na-pmSAH. (B): Funnel plots of the incidence for all studies in Meta-analysis. each point represents a separate study in the indicated association after 48studies were filled by a nonparametric trim and fill method (the solid circle represented studies which were filled). (C): Trim-and fill funnel plots were used to observe and adjust publication bias and asymmetries. (D): Linear regression test of funnel plot asymmetry (Egger test, Kolmogorov-Smirnov Z test). The intercept indicating Normal distribution is 0.2862(The intercept indicating bias is 5.80). P-value = 0.0137.

**
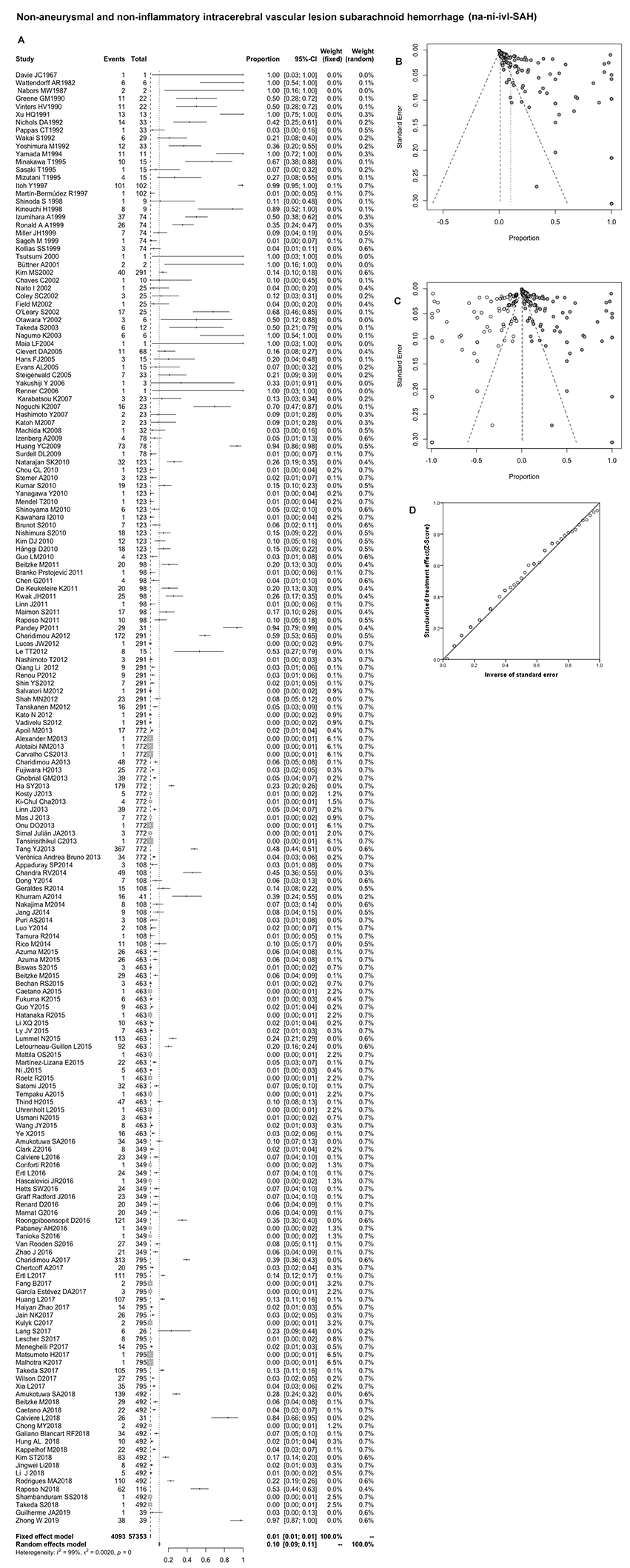
**

**Figure S9.** **Forest plots for the subgroup analysis of population–based non- aneurysmal non- non- inflammatory &intracerebral vascular lesion subarachnoid hemorrhage (na-ni-ivi-SAH) prevalence**. (A): Forest plot of the subgroup analysis by na-ni-ivl-SAH. (B): Funnel plots, each point represents a separate study in the indicated association after 184 studies were filled by a nonparametric trim and fill method (the solid circle represented studies which were filled. (C): Trim-and fill funnel plots were used to observe and adjust publication bias and asymmetries. (D): Linear regression test of funnel plot asymmetry (Kolmogorov-Smirnov Z test). The intercept indicating Normal distribution is 0.1873. P-value = 0.0049, indicating significant publication Normal distribution


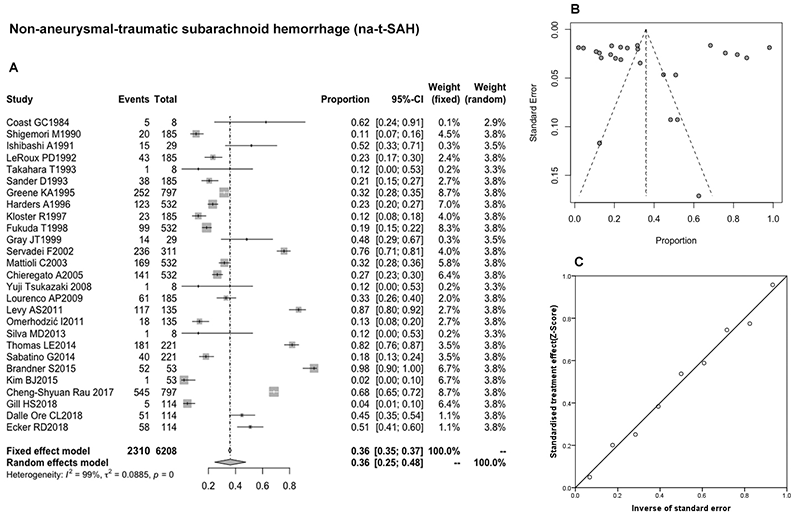


**Figure S10.** **Forest plots for the subgroup analysis of population–based non-aneurysmal subarachnoid hemorrhage coexisting with trauma (na-t-SAH) prevalence.** (A): Forest plot of the subgroup analysis by na-t-SAH. (B): Funnel plots, each point represents a separate study in the indicated association after 27 studies were filled by a nonparametric trim and fill method (the solid circle represented studies which were filled). (C): Trim-and fill funnel plots were used to observe and adjust publication bias and asymmetries. (D): Linear regression test of funnel plot asymmetry (Kolmogorov-Smirnov Z test). The intercept indicating Normal distribution is 0.3878. P-value = 0.9808, indicating significant publication Normal distribution


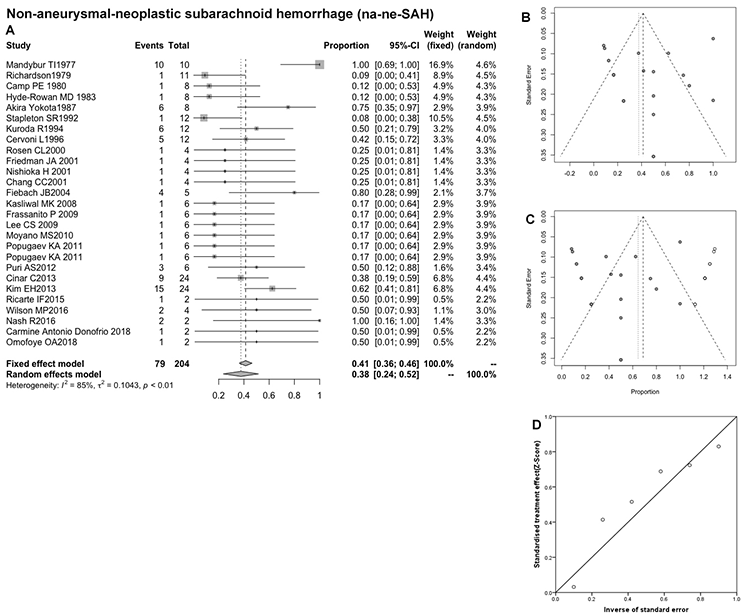


**Figure S11.** **Forest plots for the subgroup analysis of population–based non aneurysmal subarachnoid hemorrhage coexisting with neoplastic lesion (na-ne-SAH) prevalence.** (A): Forest plot of the subgroup analysis by na-ne-SAH. (B): Funnel plots, each point represents a separate study in the indicated association after 25 studies were filled by a nonparametric trim and fill method (the solid circle represented studies which were filled). (C): Trim-and fill funnel plots were used to observe and adjust publication bias and asymmetries. (D): Linear regression test of funnel plot asymmetry (Kolmogorov-Smirnov Z test). The intercept indicating Normal distribution is 0.4329. P-value = 0.5155, indicating significant publication Normal distribution


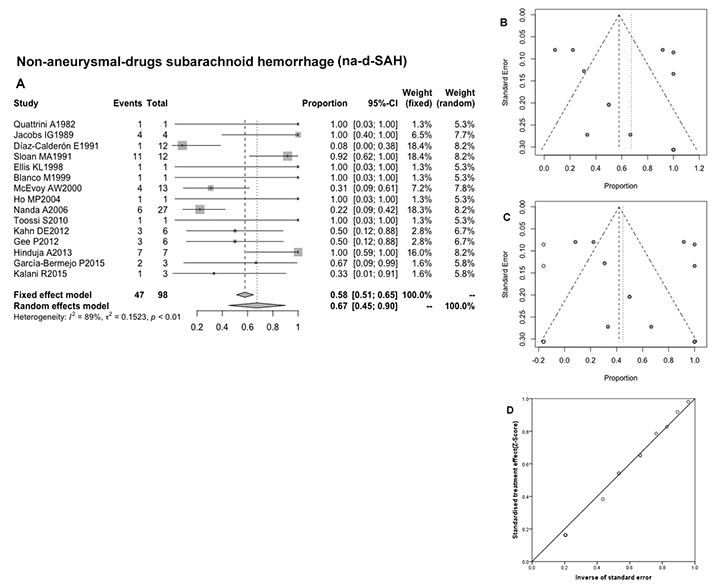


**Figure S12**. **Forest plots for the subgroup analysis of population–based non-aneurysmal subarachnoid drug hemorrhage (na-d-SAH) prevalence.** (A): Forest plot of the subgroup analysis by na-d-SAH. (B): Funnel plots, each point represents a separate study in the indicated association after 15 studies were filled by a nonparametric trim and fill method (the solid circle represented studies which were filled). (C): Trim-and fill funnel plots were used to observe and adjust publication bias and asymmetries. (D): Linear regression test of funnel plot asymmetry (Kolmogorov-Smirnov Z test). The intercept indicating Normal distribution is 0.2512. P-value = 0.0924, indicating significant publication Normal distribution
