# Supplementary material for: Novel Subgroups in Subarachnoid Hemorrhage and Their Association With Outcomes—A Systematic Review and Meta-Regression
Source: Front Aging Neurosci. 2021 Jan 11;12:573454. doi: 10.3389/fnagi.2020.573454 (PMC7829354; doi:10.3389/fnagi.2020.573454)
Supplement: Supplementary file 4 [file Data_Sheet_4.DOCX]

**Novel subgroups in Subarachnoid Hemorrhage and their association with outcomes– a systematic review and meta-regression**

*Wang, et al*

Supplementary Appendix-3.1

**Sections page**

**1) Supplementary Appendix-3. Figure.S13---**randomized controlled study (RCT) **1**

**Figure.S14** Cases cohort original studies **2**


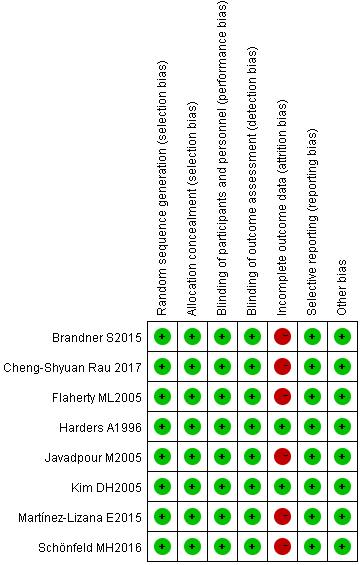


**FigureS13.** Review author’s judgements about each risk of bias item for each included RCT


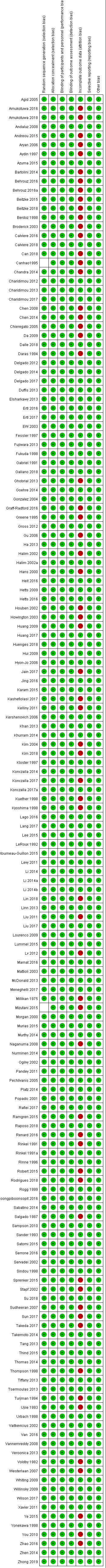


**FigureS14.** Review author’s judgements about each risk of bias item for each included Cases cohort studies
