# Supplementary material for: Novel Subgroups in Subarachnoid Hemorrhage and Their Association With Outcomes—A Systematic Review and Meta-Regression
Source: Front Aging Neurosci. 2021 Jan 11;12:573454. doi: 10.3389/fnagi.2020.573454 (PMC7829354; doi:10.3389/fnagi.2020.573454)
Supplement: Supplementary file 5 [file Data_Sheet_5.DOCX]

**Novel subgroups in Subarachnoid Hemorrhage and their association with outcomes– a systematic review and meta-regression**

*Wang, et al*

Supplementary Appendix-3.2

**Sections page**

**1) Supplementary Appendix-3 Figure.S15** cases-control original studies **1**


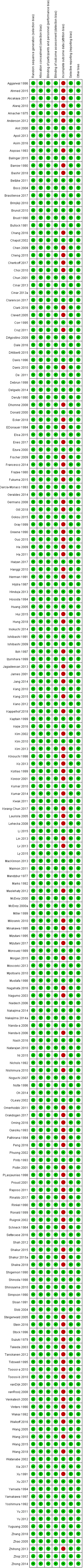


**FigureS15.** Review author’s judgements about each risk of bias item for each included cases-control studies.
