# Supplementary material for: Novel Subgroups in Subarachnoid Hemorrhage and Their Association With Outcomes—A Systematic Review and Meta-Regression
Source: Front Aging Neurosci. 2021 Jan 11;12:573454. doi: 10.3389/fnagi.2020.573454 (PMC7829354; doi:10.3389/fnagi.2020.573454)
Supplement: Supplementary file 6 [file Data_Sheet_6.DOCX]

**Novel subgroups in Subarachnoid Hemorrhage and their association with outcomes– a systematic review and meta-regression**

*Wang, et al*

Supplementary Appendix-3.3

**Sections page**

**1) Supplementary Appendix-3 Figure.S16** cases series original studies **1**





**FigureS16.** Review author’s judgements about each risk of bias item for each included cases-series report studies.
